# Design and experimental study of a precision fluid hill-drop planter for maize

**DOI:** 10.1371/journal.pone.0337887

**Published:** 2025-12-04

**Authors:** Zhengwei Zhang, Lang Zhou, Anping Ji, Zhaoran Sun, Guangyao Zhu, Xiong Chen, Junhao Pi, Chenxi Yang, Haoxuan Chen, Zhenyu Yang, Hu Tian

**Affiliations:** 1 School of Mechanical Engineering, Chongqing Three Gorges University, Chongqing, China; 2 Chongqing Engineering Technology Research Center for Light Alloy and Processing, Chongqing, China; Shahrekord University, IRAN, ISLAMIC REPUBLIC OF

## Abstract

The study presents the design of a precision maize hill-drop dibbler based on fluid control and zero-speed seeding theory, developed to overcome challenges of poor planting precision, seed damage, limited terrain adaptability, and low water-use efficiency in maize cultivation across the hilly regions of Southwest China. To this end, a novel precision fluid hill-drop planter was designed, integrating fluid control with zero-speed seeding theory. The device employs a seed-liquid separation and terminal mixing design, where a crank-connecting rod-driven piston pump ensures precise fluid delivery. A direct comparative experimental framework was established, evaluating the proposed planter against a traditional spoon-wheel seeder under identical bench-test conditions. Performance was assessed through CFD-DEM coupled simulation and systematic experiments across multiple dimensions: seeding precision (qualified, multiple, and miss index), hill-forming characteristics, and fluid performance (water application per hill, seed bounce distance). The comparative results demonstrated that within an operating speed range of 1.2 ~ 1.6 m/s, the new planter achieved a qualified index exceeding 91%, a significant improvement of 12.5% over the conventional device. The seed bounce distance was controlled within 5.4 mm, representing a 63.2% reduction. Furthermore, the system exhibited excellent operational stability, with a coefficient of variation for water application per hill of less than 2% and a check valve leakage rate below 3%. Through collaborative parameter optimization, breakthrough indicators were achieved: a 94.8% seed-water coincidence rate and a hill spacing deviation of no more than 1.0%. This research validates the proposed planter’s superior performance and reliability, providing an effective technical solution to enhance sowing uniformity and water-use efficiency in complex terrain.

## 1. Introduction

Corn, as one of China’s most important grain crops, reached a total production of 29.49 million tons in 2024, accounting for approximately 40% of the nation’s total grain output [1]. In southwestern China, it holds significant strategic importance for local agricultural production and regional economic development [2,3]. As a global leader in corn production, the United States is often cited as a benchmark for large-scale, highly mechanized agriculture, with a national average corn yield of 11.27 t/ha [4]. However, China’s corn productivity significantly lags, with its average yield being only 6.59 t/ha. This gap is further evidenced when compared with other major producers such as Argentina, where average yields reach 7.21 t/ha with high-yielding regions achieving up to 9.39 t/ha. Notably, China’s own yield potential is demonstrated by the record of over 15 t/ha achieved through intensive planting technologies in Xinjiang, highlighting the significant potential for improving efficiency through advanced sowing technologies and scaled production [5]. The substantial productivity gap is attributed to fundamental structural and technological differences, including smaller farm sizes, lower mechanization levels, and particularly, key constraints in sowing technology [6]. Traditional corn planting methods suffer from insufficient precision and high seed loss, while existing mechanical sowing equipment struggles to adapt to the complex terrain conditions of hilly regions [7,8]. Fluid sowing technology, by integrating water-assisted sowing techniques, can enhance seed bed stability, improve seedling emergence rates, and reduce water consumption, offering a promising solution to current sowing challenges [9,10].

Significant research advancements have been made in precision seed metering technology globally. Internationally, Richard et al. developed a narrow-row vertical disc seed metering device [11], while Hunt et al. designed a plunger-type precision vegetable seeder [12]. In China, research has exhibited a diversified development trend. For instance, Lin et al. optimized segmented maize sowing based on the SLD model [13]; Du et al. designed a self-disturbance internal-filling hole-wheel maize precision seed metering device [14]; Xing et al. conducted experimental studies on an orientation-pushing device for dent corn seeds [15]; and Zhao Shuhong et al. optimized maize planter furrow openers using the discrete element method [16]. Concurrently, in fluid seeding technology, Chinese researchers have developed innovative designs such as a siphon-based maize fluid hill-drop device [17], a dual-disc wheel-type precision hill-drop seeder [18], and pump-driven seed metering systems [19,20].

Despite these developments, a critical research gap remains in effectively integrating high-precision seed metering with robust fluid delivery systems specifically for the undulating and fragmented terrain of Southwest China. Traditional mechanical seeders often lack the necessary adaptability and seeding accuracy on slopes. Although fluid seeding technology shows potential, its operational performance in hilly areas—particularly concerning stability on slopes and the uniformity of the seed-liquid mixture—is not yet fully validated or optimized. This highlights a clear need for a planting solution that synergistically combines precision, terrain adaptability, and resource efficiency.

In recent years, advanced computational methods have provided powerful tools for addressing such complex engineering challenges. The application of machine learning (ML) and explainable artificial intelligence (XAI) in agricultural engineering, as demonstrated in studies on drying processes [21], biomass gasification [22], and nanofluid systems [23], offers valuable methodologies for parameter optimization and system interpretation. Furthermore, dynamic control strategies from other domains, such as amphibious vehicle control [24], provide relevant insights for designing adaptive agricultural machinery.

To bridge this technological gap, this paper proposes a novel fluid-controlled precision hill-drop planter based on zero-speed seed delivery theory [25,26]. which aims to nullify the seed’s horizontal velocity at the moment of contact with the seedbed to achieve precise deposition and minimize bounce. The core innovation lies in the integration of a crank-link-driven piston pump for precise fluid delivery and a seed-guiding device designed to minimize the seed’s horizontal velocity now of release. Key innovations include a pressure gradient model that reduced seed collision energy loss by 63.2% and a crank-link-driven piston pump with a DN80 pipeline that reduced head loss by 42%. Bench tests confirmed that the proposed device significantly enhances terrain adaptability and water-use efficiency compared to traditional seeders. This study not only addresses the core limitations of existing sowing technologies in complex terrains but also provides a practical and efficient solution for advancing maize planting mechanization in China.

## 2. Overall structure and working principle

**Fig 1:**
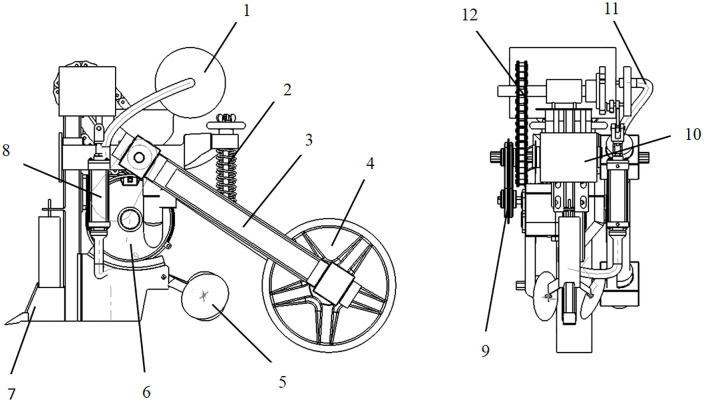
Structure of the whole machine. 1. Water tank; 2. Damper; 3. Coupling; 4. Ground wheel; 5. Soil covering device; 6. Seed metering device; 7. Furrow opener; 8. Water-supplying mechanism; 9. Sprocket housing; 10. Frame; 11. Hose; 12. Sprocket.

As shown in Fig 1, the precision fluid hill-drop corn planter mainly consists of a fluid control mechanism, seed metering device, furrow opener, and press wheel.

The hill-drop planter operates through coordinated tractor traction and spring-based profiling mechanisms. When the furrow opener penetrates the soil under the gravitational force of the seeding unit, the drive mechanism transmits ground wheel rotation to the seed metering shaft and water-delivery mechanism via bevel gears and sprocket systems. This actuates piston downward movement to compress the sealed chamber, simultaneously closing the inlet valve through a hinged flap mechanism while opening the outlet valve for precise water application.

Concurrently, corn seeds from the hopper are simulated by a side-filling seed metering device with agitating elements, temporarily stored in a seed tube equipped with flexible baffles and subsequently mixed with the water flow for combined seed-liquid delivery. The press wheel completes the soil covering and compaction process. This integrated system achieves precise synchronization of seeding and irrigation through mechanical hydraulic linkage [27].

## 3. Design of key components

### 3.1 Fluid control mechanism

**Fig 2:**
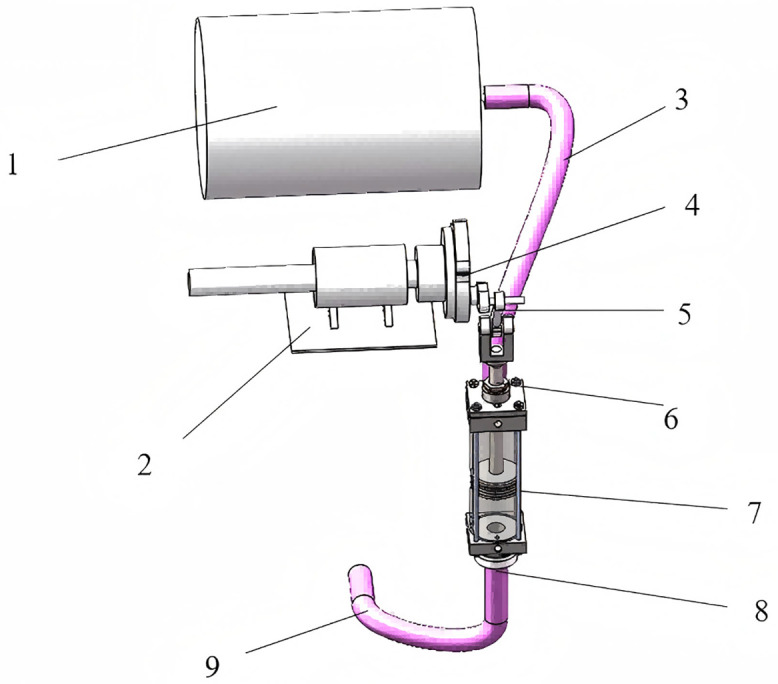
Fluid control mechanism. 1. Water tank; 2. Bearing housing; 3. Water pipe; 4. Crank disk; 5. Connecting rod; 6. Piston rod; 7. Piston; 8. Nozzle; 9. Water pipe.

As shown in Fig 2, the fluid control mechanism consists of a gravity water supply tank, a crank-connecting rod drive unit, and a DN80 standard piping system. The water tank is used to provide gravitational potential energy, while the crank connecting rod drive unit converts the rotational motion of the crank plate into the linear reciprocating motion of the piston. Through the synergy between the reciprocating motion of the piston and the low-loss pipeline design, accurate flow control is realized while ensuring efficient utilization of water resources.

#### 3.1.1 Hydraulic modeling and parameter optimization.

**Fig 3:**
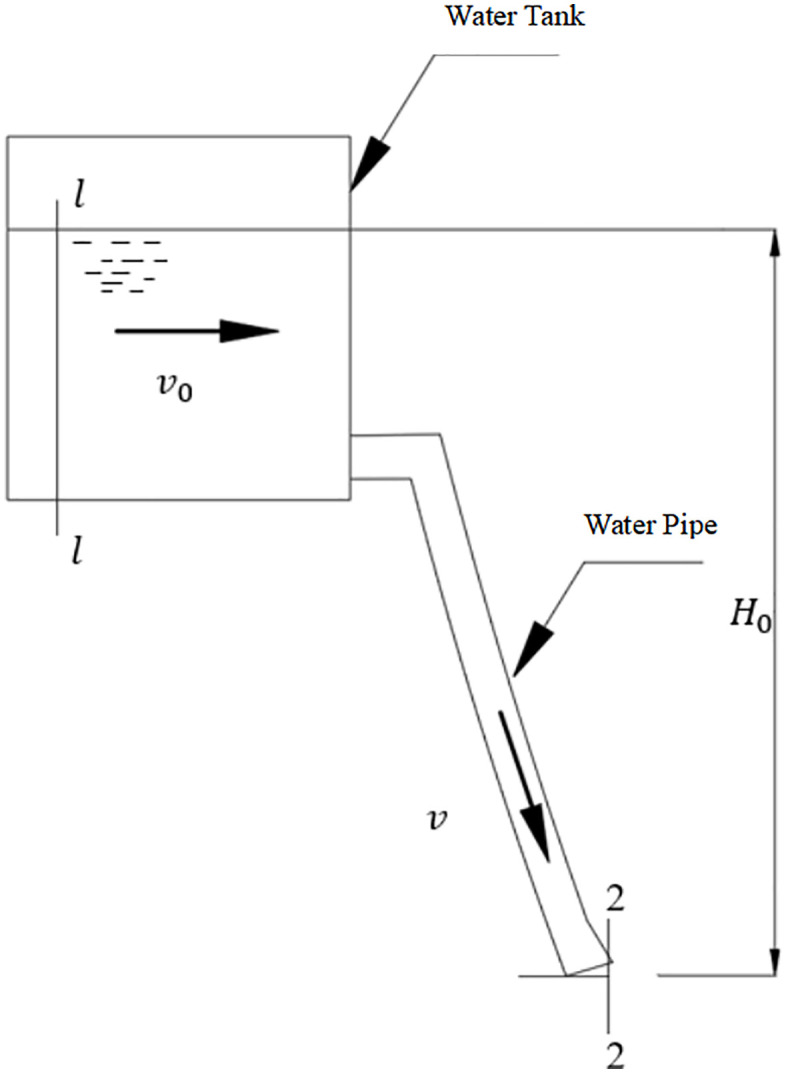
Flow pipeline diagram.


Fig 3 presents a simplified flow circuit diagram of the fluid control mechanism, The flow characteristics can be described by a modified Bernoulli equation. Taking the outlet plane (0−0) as the reference datum, and considering a tank hydrostatic head (*H*) of 0.5 m and a turbulent energy correction coefficient (α) of 1.0624, the energy conservation equation is established as follows [28]:

$$H+\frac{P_{a}}{\gamma }+\frac{\alpha_{0}{\upsilon_{0}}^{2}}{2g}=\frac{\alpha \upsilon^{2}}{2g}+h_{w}$$ (1)

Where H is the still water head of the tank (m), Pa is the atmospheric pressure (Pa), α is the turbulence correction coefficient (1.06), γ is the bulk density of water (N/m³), $\upsilon_{0}$ is the falling speed of the tank liquid level (m/s), υ is the flow velocity at the outlet of the pipeline (m/s), g is the gravitational acceleration (9.81 m/s^2^), and $h_{w}$ is the total head loss (m).

The total head loss $h_{w}$ is composed of the loss along $h_{f}$ and the local loss $h_{j}$:


$$h_{w}=h_{f}+h_{j}$$ (2)

The Darcy-Weisbach equation is used for the loss along the path:


$$h_{f}=\lambda \frac{l}{d}\frac{\upsilon^{2}}{2g}$$ (3)

Where λ is the drag coefficient along the way, *l* is the total *l*ength of the pipeline, and d is the internal diameter of the pipeline.

λ = 0.316Re^-0.25^ = 0.032 calculated by Blasius formula, according to standard local loss [29]:


$$h_{j}=\sum \zeta \frac{\upsilon^{2}}{2g}$$ (4)

where ∑ζ is the sum of local drag coefficients.

Local resistance coefficient is ∑ζ 0.9: composed of inlet resistance coefficient 0.5, outlet resistance coefficient 0.3 and elbow resistance coefficient 0.1. The expression for total head loss is:


$$h_{w}=\left(\lambda \frac{l}{d}+\sum \zeta \right)\frac{\upsilon^{2}}{2g}$$ (5)

Substituting equation (5) into equation (1), flow coefficient u and volumetric flow *Q* are derived:


$$u=\frac{\text{1}}{\sqrt{1+\lambda \frac{l}{d}+\sum \zeta }}$$ (6)

$$Q=uA\sqrt{2gH_{0}}$$ (7)

where Q is the volumetric flow rate (m^3^/s), $H_{0}$ is the total applied head (1.0 m), μ is the pipe discharge coefficient (0.337), and A is pipe cross-sectional area (4.91104 m^2^).

**Fig 4:**
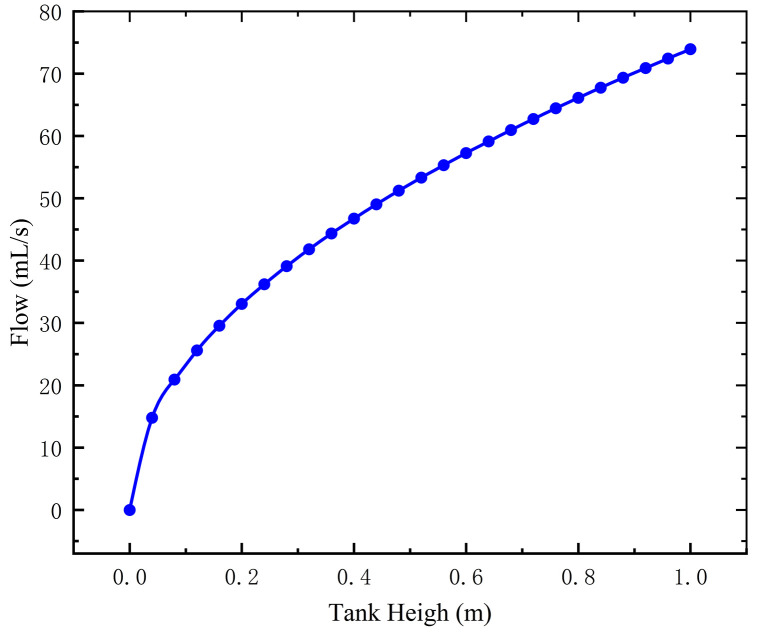
Flow vs. tank height.

Substituting the resistance characteristic into equation (6), the flow coefficient is 0.337 and the flow Q of equation (7) is 73.9 mL/s. The experimental results show that the calculated values are in good agreement with the measured values of 71.2 ± 3.5 mL/s. The parameter sensitivity analysis shows that when the water head $H_{0}$ decreases from 1.0 m to 0.2 m, the flow Q decreases by 55.3%. This trend is consistent with the flow-water level relationship curve shown in Fig 4.

Both theoretical analysis and experimental verification indicate that in traditional gravity-fed irrigation systems, the flow rate (Q) is highly sensitive to changes in the tank’s hydrostatic head (H) In contrast, the present design utilizes a crank-link-driven piston pump as the dominant component for flow control. Its fixed-displacement characteristics significantly reduce the system’s sensitivity to variations in water tank height, which is a key advantage of our design. This advantage ensures stable operational flow rates within the practical tank height range of 1.2 ~ 1.5 m, as will be experimentally validated in Section 5.2.2.

To ensure sufficient and stable water flow, the diameter of the water pipe needs to be optimized. Based on the theory of circular hole outflow, the optimal diameter d of flow is:


$$d=\sqrt{\frac{4Q}{\pi \mu \sqrt{2gH}}}$$ (8)

Where H is the head height (m).

The optimal pipe diameter d = 27.08 mm is calculated by equation (8), but the actual DN80 standard pipe (80 mm) is selected to take into account the processing tolerance and 20% flow design redundancy ($Q_{\max}$ =150 mL/s). After optimization, the total efficiency of the system increased by 41.2%.

### 3.2 Seed-metering device

**Fig 5:**
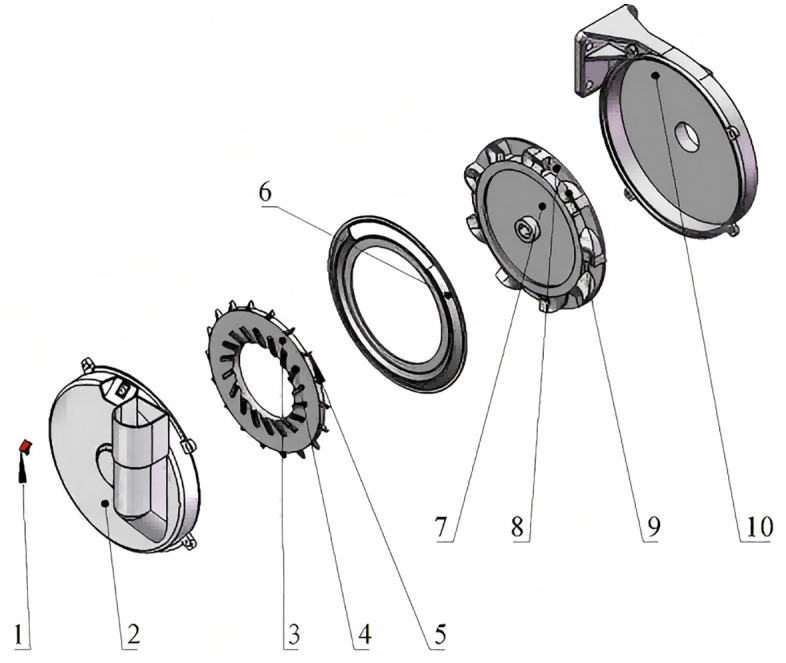
Model of the seed-metering device. 1. Seed cleaning brush; 2. Transparent housing; 3. Seed plate; 4. Seed agitating strip; 5. Seed spoon; 6. Dividing septum; 7. Seed conveying disk; 8. Seed delivery orifice; 9. Seed-filling curved surface; 10. Seed hopper.

**Fig 6:**
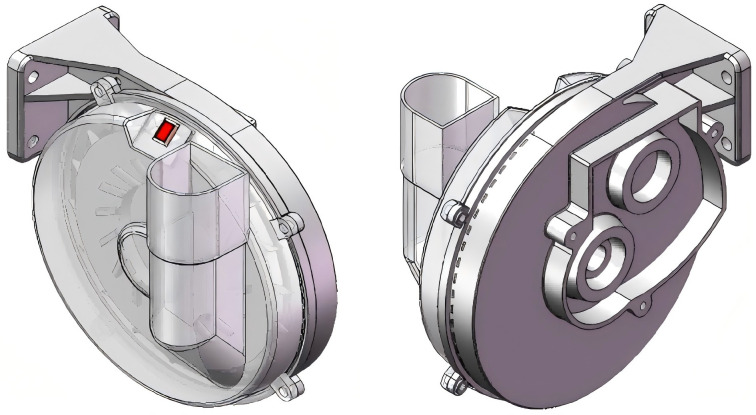
Arrangement of the seed agitating strips on the metering disc.

As illustrated in Fig 5, the seed metering device employs an improved spoon-wheel design, primarily comprising a seed metering disc, a transparent housing, an intermediate partition plate, a seed-filling chamber, a seed conveying disc, and a seed-cleaning brush. Unlike conventional vertical disc-type seed plates, this metering device features a spoon-shaped seed pickup structure positioned along the periphery of the metering disc. The seed metering disc, conveying disc, and main shaft are rigidly connected and driven by an external power source. The intermediate partition plate isolates the seed discharge area from the conveying tray, while agitation ribs are arranged around the metering disc to directly disturb seeds, thereby enhancing seed-filling performance, as depicted in Fig 6.

#### 3.2.1 Seed plate.

**Fig 7:**
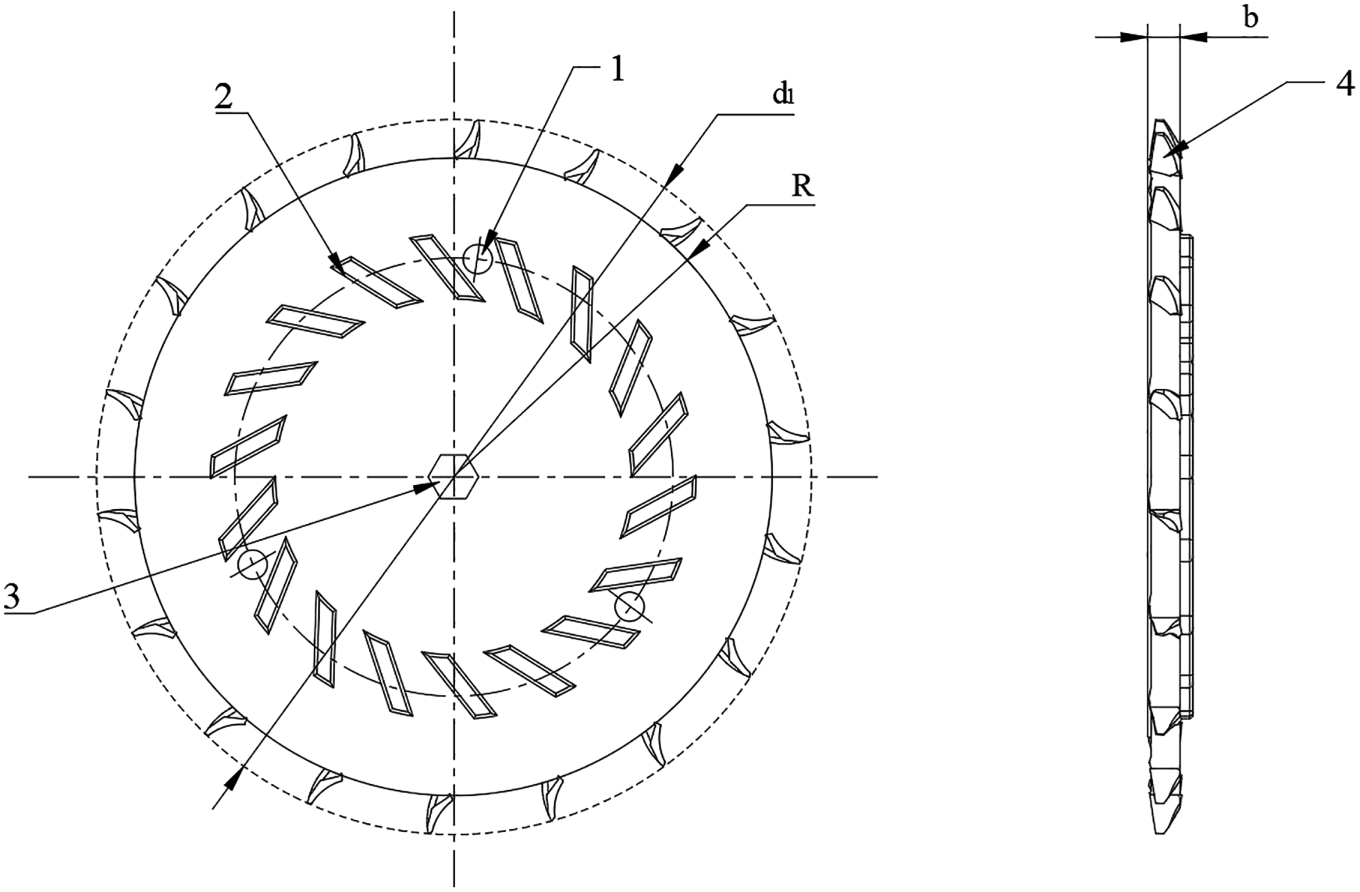
Structure of the seed-metering plate. 1. Mounting screw hole; 2. Seed agitating rib; 3. Central shaft; 4. Seed cell with spoon profile.

The seed-filling plate is the core component of the seed-metering device, primarily responsible for seed pickup, seed clearing, and seed delivery. With reference to Agricultural Machinery Design Handbook, a seed-filling plate with a diameter of 200 mm was adopted to ensure optimal seed pickup and clearing performance. Additionally, the design of the seed-disturbing strips was optimized to effectively reduce seed damage and improve seed-filling efficiency, as illustrated in Fig 7. The structural parameters of the seed-filling plate are as follows: The seed-scooping disk is equipped with three M6 through holes, with an included angle of 120° between adjacent holes and a 20° angle between the holes and the seed-scooping units.

**Fig 8:**
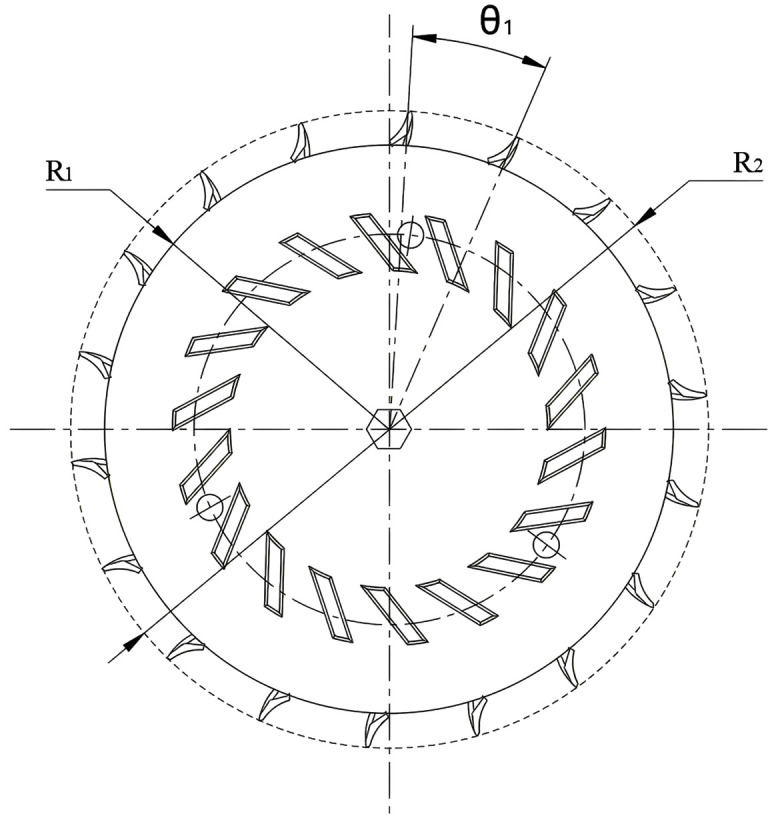
Geometric structure and parameters of the seed scoop wheel.


The number of seed scoops and the size of the seed scoop wheel are key factors in calculating the seeding speed and seeding distance as shown in Fig 8. The formula for calculating the number of seeds is:

$$Z_{1}=\frac{360^{\circ }}{\theta_{1}}$$ (9)

Where $Z_{1}$ is the number of seed scoops and $\theta_{1}$ is the angle between two adjacent seed scoops and the center line of the seed scooping wheel.

**Fig 9:**
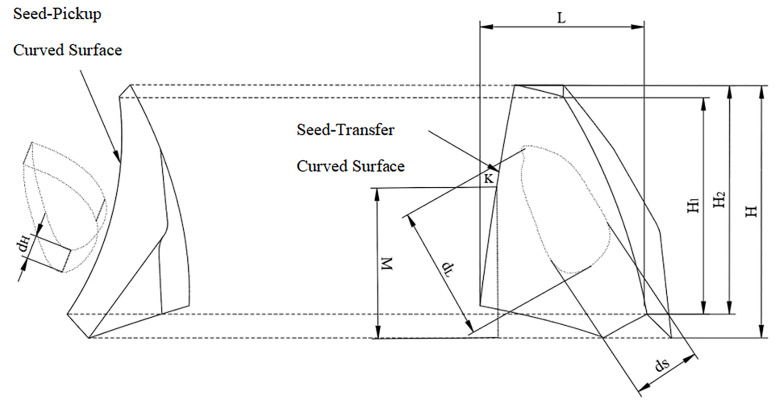
Structure of the seed metering spoon. Front View. Right View.


The inner radius $R_{1}$ of the seed spoon hole is 83 mm, and the outer diameter $R_{2}$ of the seed spoon wheel is 115 mm. By calculating the number of seed scoops $Z_{1}$ is 18. According to the average size of corn seeds (length: 10.13 mm, width: 9.24 mm, thickness: 7.36 mm), the spoon structure was obtained by simplifying the design as shown in Fig 9 The hole design of the seed metering spoon shall ensure that the seeds are stable and can pass through the seed metering device smoothly. For seeds to fill the spoon hole smoothly, it is necessary to satisfy:

$$\left\{ \begin{array}{c} H=2d_{L}+k_{L}\\ H>H_{2}>H_{1}\\ d_{s}\leq L\leq 1.5d_{s}\\ 0.5d_{L}\leq H\leq 1.5d_{L} \end{array}\right.$$ (10)

Where H is the total longitudinal width of the groove (mm), $d_{L}$ is the average length of corn seeds (mm), $k_{L}$ is the length value (2 ~ 4 mm), $H_{1}$ is the longitudinal width of the seed taking arc surface (mm), $H_{2}$ is the longitudinal width of the arc surface (mm), $d_{s}$ is the average width of corn seeds (mm).

To ensure stable seed injection, the following parameters are set: H = 15 mm, $H_{1}$ = 11 mm, $H_{2}$ = 12 mm, and L = 8.2 mm. The vertical distance (M) from the shrinkage point to the groove end surface is required to be greater than the seed length of 13 mm. The seed insertion angle is set at 60°.

#### 3.2.2 Dynamic analysis of maize kernels.

1. Force Analysis During Seed Pick-up

**Fig 10:**
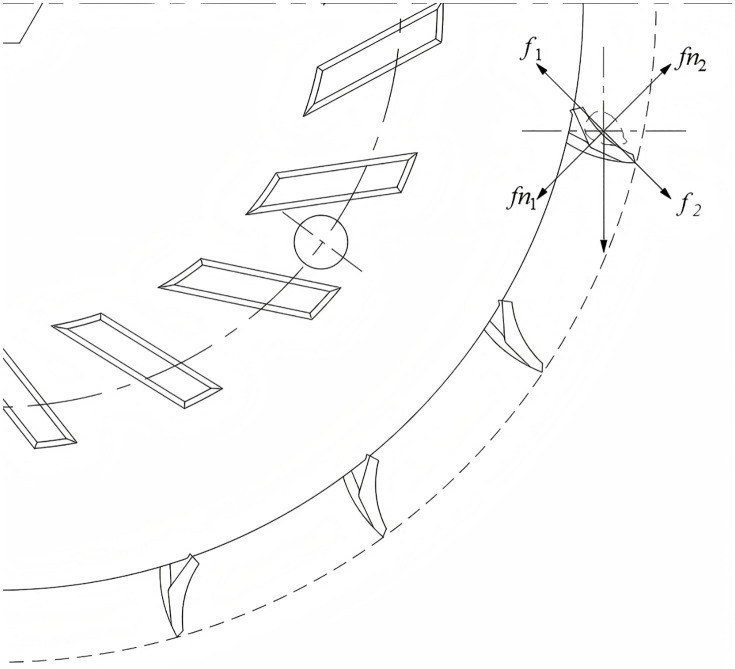
Force analysis of seed during the seed picking process.

During the seed pickup process, the seed undergoes uniform circular motion within the seed spoon. The force analysis of the seed during the acceleration of the seed pickup device is illustrated in Fig 10.

The equilibrium equations of corn seed forces during the seed picking stage are established as follows:


$$\left\{ \begin{array}{c} f_{2}=F_{n2}\tan\varphi_{2}\\ f_{1}=m\omega^{2}R-mg\sin\theta \\ F_{n1}=mg\cos\theta +f_{2} \end{array}\right.$$ (11)

Where $f_{2}$ is the static friction force (N) between the grain and the grain guide table on the seed metering device side, $f_{1}$ is the thrust force (N) of the seed spoon, $F_{n2}$ is the sliding friction force (N) between the grain and the partition plate, $\varphi_{2}$ is the static friction angle between the grain and the groove of the feeding wheel, and $F_{n1}$ is the support force (N) of the partition plate on the grain.

To prevent damage to the seed pickup device, the maximum acceleration during the seed pickup process must not exceed $a_{1}$:


$$a_{1}=\frac{\omega^{2}R-g\sin\theta }{\tan\varphi_{1}}$$ (12)

Where R is the radius of rotation (mm) and the sliding friction angle (°) between the seed and the separator.

2. Force Analysis During Seed Cleaning

**Fig 11:**
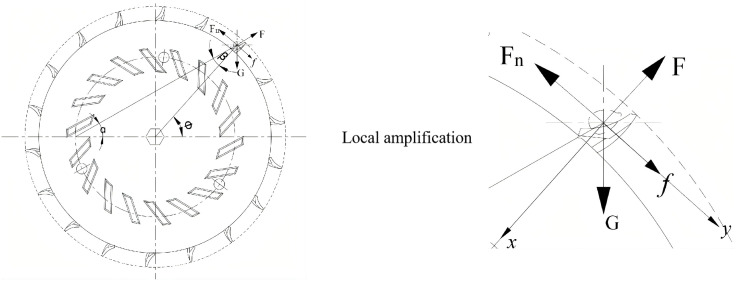
Stress analysis of seeds during seed cleaning.

During seed cleaning, excess seeds are expelled from the seed spoon by centrifugal force and friction. The kernel is subjected to gravity, centrifugal force and friction force, as shown in Fig 11.

According to the movement of seeds, the equilibrium equation of force is established:


$$\left\{ \begin{array}{c} G_{1}=mg\sin\alpha \\ F_{1}=m\omega^{2}r\cos\beta \\ F_{2}=m\omega^{2}r\sin\beta \end{array}\right.$$ (13)

For the seeding device to seed smoothly, $G_{1}$ should be greater than the sum of $F_{1}$ and f Therefore, the limit acceleration $a_{2}$ in the seeding process is incorporated into the above equation (13). From the relationship of trigonometric function:


$$\left\{ \begin{array}{c} \sin\alpha =\frac{r\sin\alpha }{\frac{r}{\cos\beta }}=\cos\beta \sin\phi \\ {}[6pt]\cos\alpha =\sqrt{1-\cos^{2}\beta \sin\phi } \end{array}\right.$$ (14)

Solving equation (14) yields $a_{2}$ as:


$$a_{2}=\frac{\left(\cos\beta \sin\phi -\mu \sqrt{1-\cos^{2}\beta \sin\phi }\right)g+\omega^{2}r\left(\mu \sin\beta -\cos\beta \right)}{\mu }$$ (15)

From equation (15), it can be seen that the acceleration in the seed cleaning process is not exceeded $a_{2}$, and the phenomenon of “over-cleaning” can be avoided.

3. Force Analysis During Seed Conveying and Release

**Fig 12:**
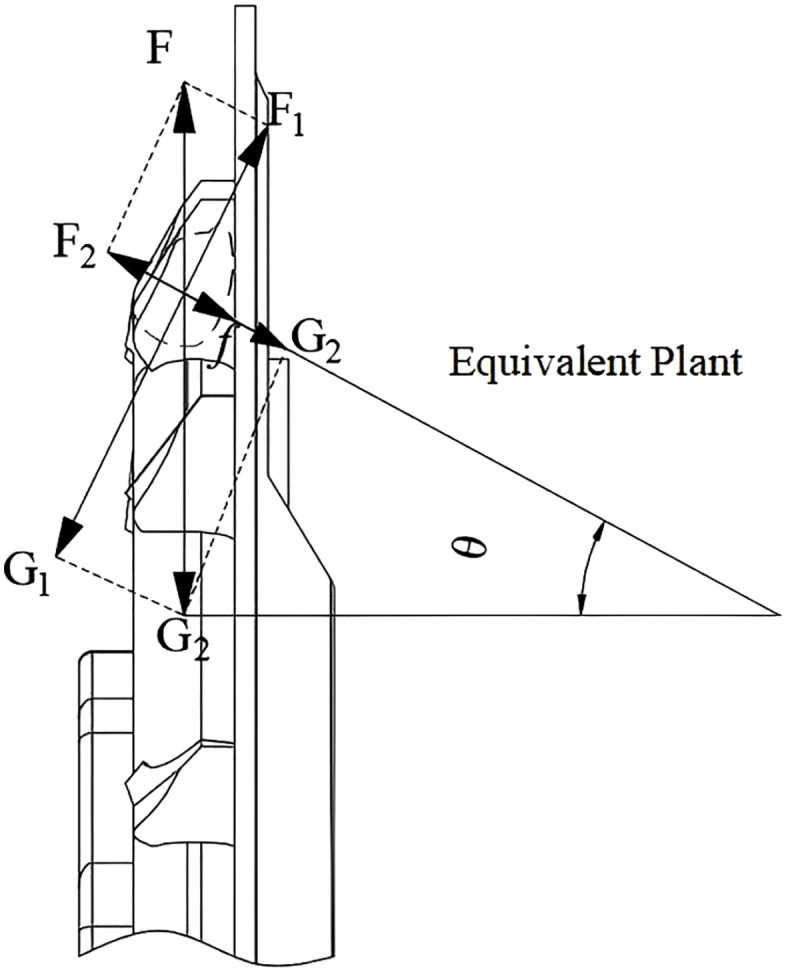
Stress analysis of seed propagation process.

During the seed transmission process, the seed spoon carries seeds into the seed delivery area and deposits them into the tray through the delivery port [30]. This process can be simplified into an equivalent inclined plane model, as illustrated in Fig 12.

Consider the seed being subjected to the gravitational force G, centrifugal force F, and frictional force f. These forces are decomposed into components along the direction of the seed delivery hole ($G_{1}$) and the direction acting on the seed metering device ($G_{2}$).


$$\left\{ \begin{array}{c} G_{1}=mg\cos\theta \\ G_{2}=mg\sin\theta \\ F_{1}=m\omega^{2}r\cos\theta \\ F_{2}=m\omega^{2}r\sin\theta \\ f=\mu \left(G_{1}-F_{1}\right) \end{array}\right.$$ (16)

**Fig 13:**
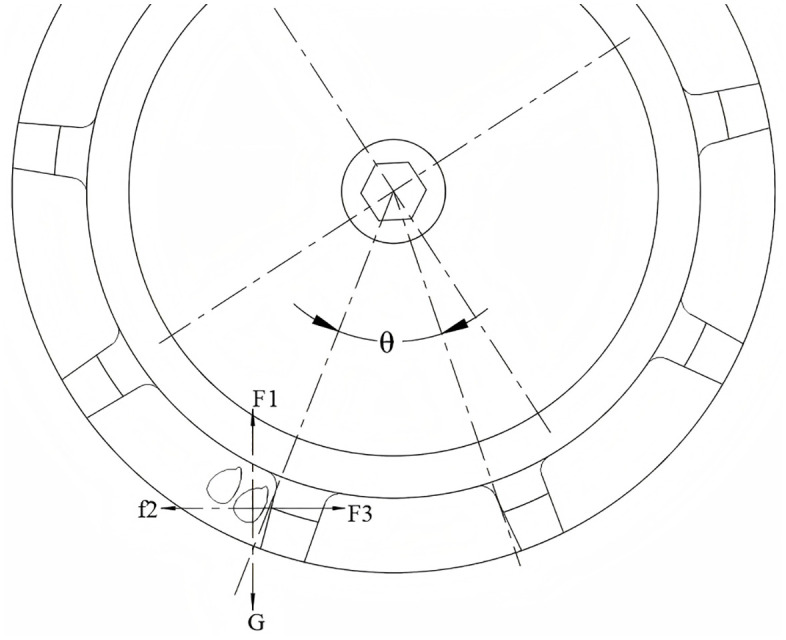
Force analysis during the seed feeding process.

During the seed feeding and dispensing process in a maize seeding system, the kernel is transported from the seed spoon to the delivery plate and finally to the seed outlet. The force analysis of the seed’s motion during this process is illustrated in Fig 13.

Based on the dynamic analysis, it can be concluded that:


$$\left\{ \begin{array}{c} G-F_{1}=0\\ F_{3}-f_{2}=0 \end{array}\right.$$ (17)

Where G is the gravity (N) on the corn kernel itself, $F_{3}$ is the pushing force (N) on the corn kernel by the side wall of the seed wheel, and $f_{2}$ is the friction force (N) on the corn kernel by the seed shell.

As illustrated in Fig 13 and supported by (17) theoretical analysis, during the later stage of seeding, the gravity of the seed, supporting forces, and frictional forces collectively position the seed within the alternating seed delivery gaps. Ultimately, during the conveying and seed release processes, the frictional force and pushing force reach equilibrium, ensuring smooth and precise seed discharge [31].

### 3.3 Seed guide device structural design

**Fig 14:**
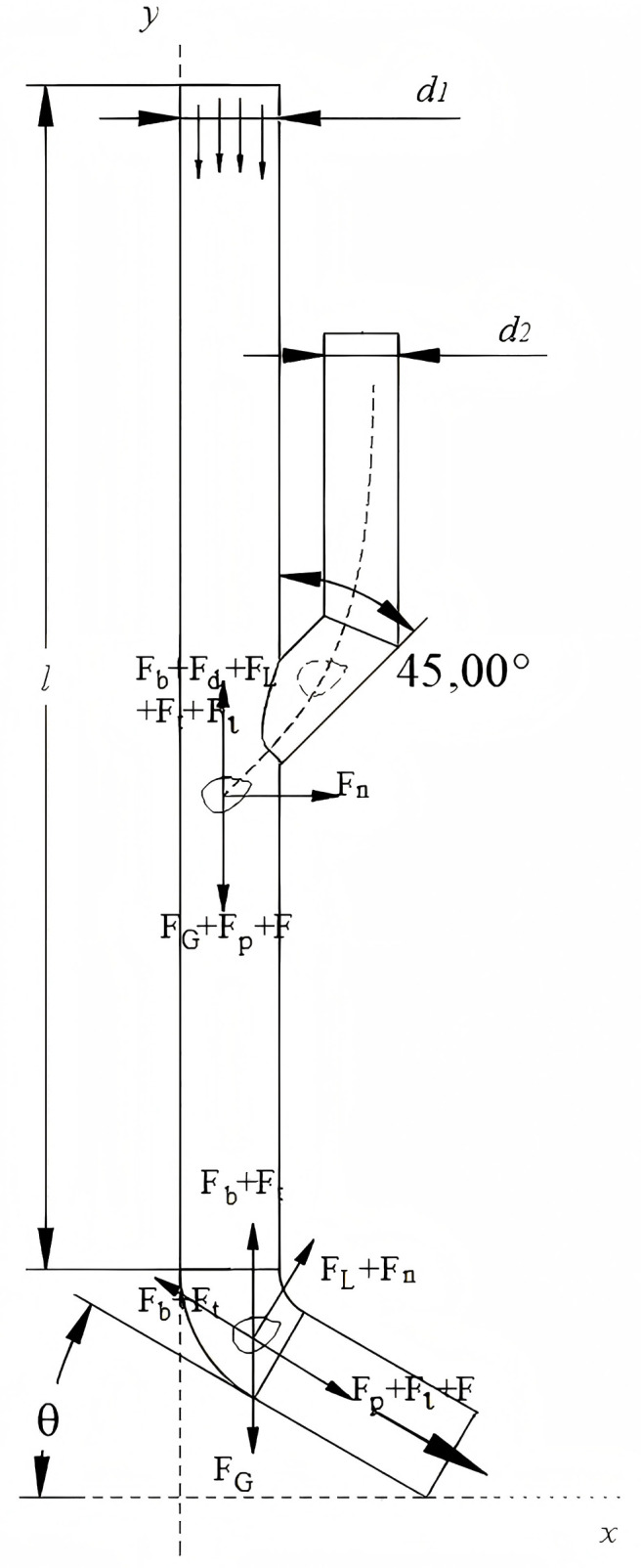
Seed guide tube. 2 D seed delivery tube.


The seed guide device is engineered based on the zero-speed seed delivery mechanism. The core objective of this mechanism is to ensure that the seed’s horizontal velocity component is nullified upon contact with the seedbed, thereby achieving precise deposition and minimizing seed bounce and displacement. As shown in Fig 14, the structural design incorporates a gradually expanding tube combined with a large bending radius, supplemented by a low-friction coating surface treatment. This integrated design effectively reduces collisions between seeds and the tube wall, dissipating in kinetic energy and guiding the seed towards a near-zero horizontal velocity at the outlet. In terms of operational parameter regulation, precise adjustment of the seed tube inclination angle and fluid velocity is crucial to realize this zero-speed delivery condition [32,33]. The particle motion characteristics within this system can be described by the following equilibrium equation:

$$m_{p}=\frac{d\mu_{p}}{dt}=F_{G}+F_{d}+F_{b}+F_{p}$$ (18)

Where $F_{G}$ is gravity, $F_{d}$ is drag, $F_{b}$ is buoyancy, $F_{p}$ is pressure gradient.

**Fig 15:**
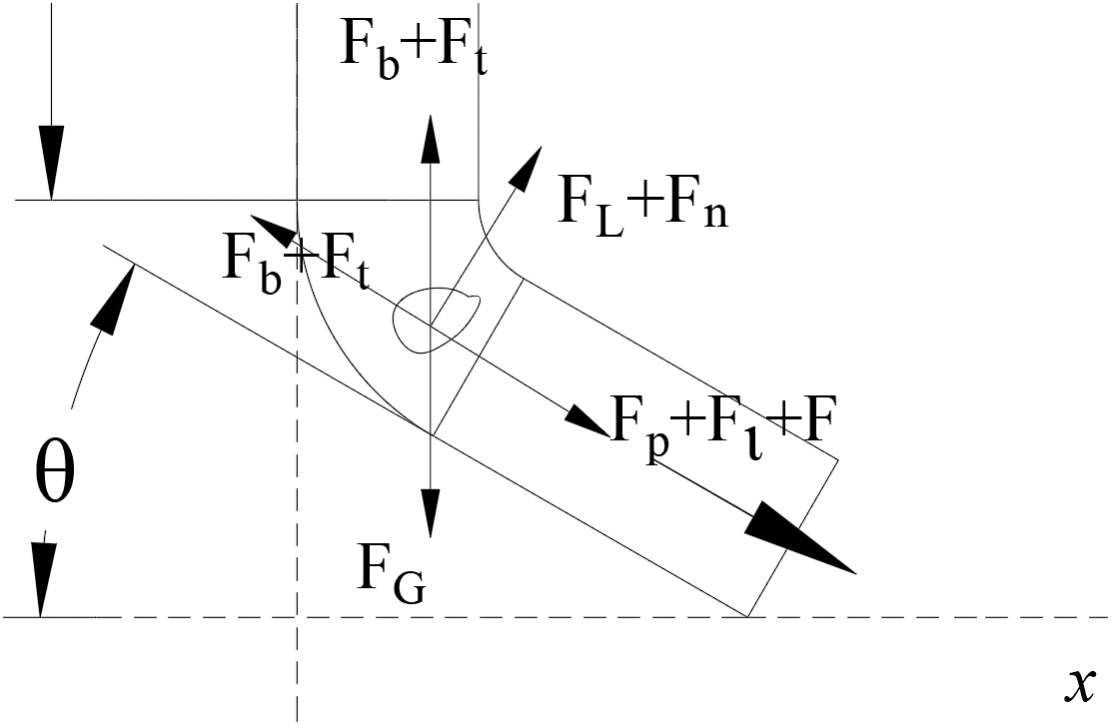
Force analysis of seed particles.


The force acting on the seed in the seed guide tube is shown in Fig 15. It is mainly dominated by drag force, effective gravity and pressure gradient force. Newton’s law is used to describe its motion:

$$mv-mv_{0}=\int_{0}^{t}\left(F_{p}+mg\right)dt-\int_{0}^{t_{1}}F_{f}dt$$ (19)

Where m is represents the mass of the seed (kg),v is the ejection velocity of the seed (m s^-1^) [34], $v_{0}$ is the initial velocity of the seed (m s^-1^), t is the duration of water flow action on the seed (s), $t_{1}$ is the contact time between the seed and the end of the seed-guiding device (s), and $F_{f}$ is the frictional force between the seed and the end of the seed-guiding device (N) [35].

The derived horizontal ejection velocity of the seed is:


$$\nu_{x}=\frac{\left(C_{d}pv^{2}A+mg\right)t-mg\cos\theta t-mg\cos\theta t_{1}}{m}+v_{0}\cos\theta$$ (20)

According to Equation (20), the seed ejection velocity is related to the inclination angle of the seed guide tube and the fluid velocity. To avoid clogging, the fluid velocity must be greater than the seed settling velocity. In subsequent simulations, comparative analyses were conducted by setting θ to 15°, 30°, and 45°, respectively.

## 4. Simulation experiments and analysis

### 4.1 Fluid control mechanism

To investigate the distribution characteristics of the fluid control mechanism in gas-liquid two-phase flow, numerical simulations were conducted using Fluent software. By simplifying the fluid control mechanism illustrated in Fig 2, the study aims to elucidate and predict fluid dynamic behavior within the piston pump. The CFD approach was employed to accurately capture the complex gas-liquid two-phase flow, pressure gradients, and energy dissipation characteristics inside the piston pump, which are crucial for understanding the delivery mechanism and optimizing system performance.

#### 4.1.1 Dynamics analysis of piston at top and bottom dead centers.

Simulation Parameter Settings the Realizable k-ε turbulence model was adopted, and the pressure-velocity coupling used the SIMPLE algorithm. The inlet boundary condition was set as a pressure inlet, and the outlet was a pressure outlet. The mesh number was 850,000 with a mesh quality Skewness < 0.75, meeting the computational accuracy requirements. The time step was set to 0.001 s, and the convergence criterion was a residual less than 10^−4^.

**Fig 16:**
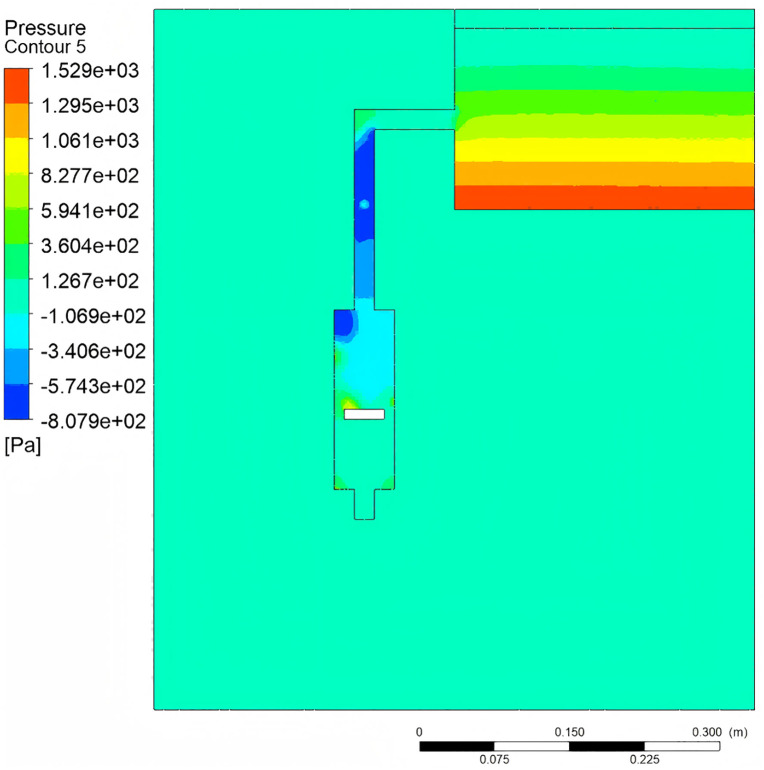
Pressure contour.

**Fig 17:**
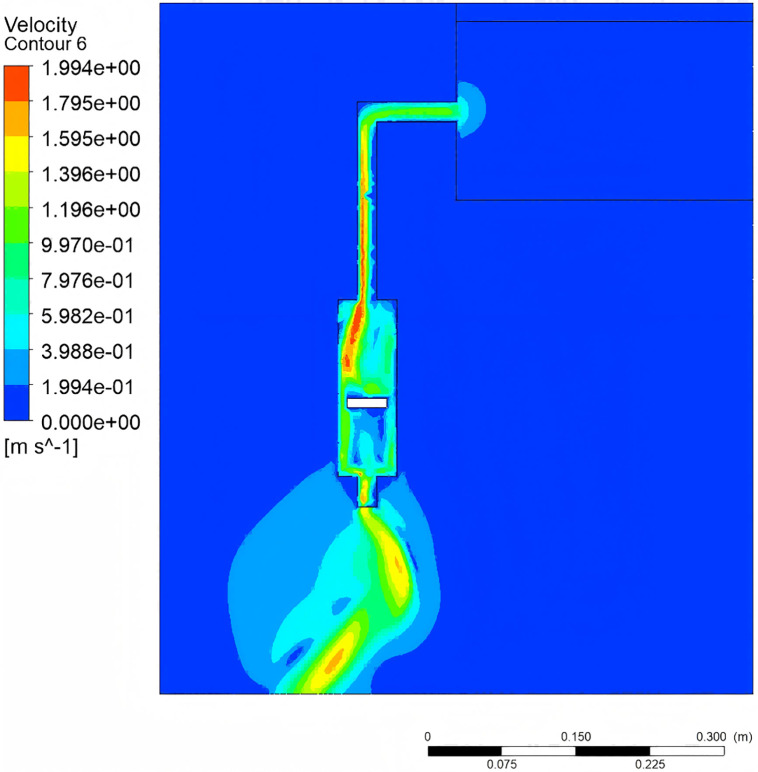
Velocity contour.

**Fig 18:**
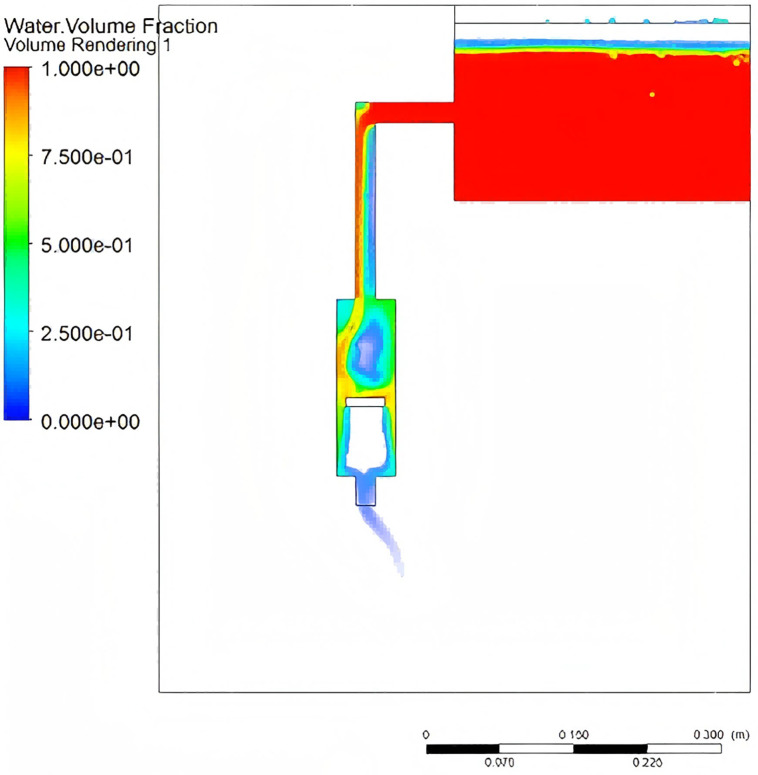
Volume fraction contour.

The pressure gradient distribution, flow velocity versus energy dissipation, and multiphase flow characteristics are shown in Figs 16–18, respectively.. As shown in Fig 16 when the piston reaches bottom dead center, a pronounced axial pressure gradient emerges within the water tank, with static pressure at the bottom exceeding that at the top. The converging pipe section exhibits pressure reduction due to cross-sectional area contraction. Fig 17 demonstrates the relationship between flow velocity and energy dissipation. The minimal flow area at the valve-pipe junction yields a peak velocity of 1.994 m/s. While the optimized nozzle design enhances outlet velocity, it may concurrently induce hydraulic shock and cavitation. Turbulent effects cause energy dissipation in the downstream section, reducing the standard deviation of pressure fluctuations. Fig 18 reveals multiphase flow features, where phase volume fraction is governed by gravity, pipe geometry, vortex motion, and cavitation effects. The piston geometry critically influences flow guidance. These findings highlight the complexity of flow patterns and pressure fluctuations, providing pivotal insights for optimized system design.

**Fig 19:**
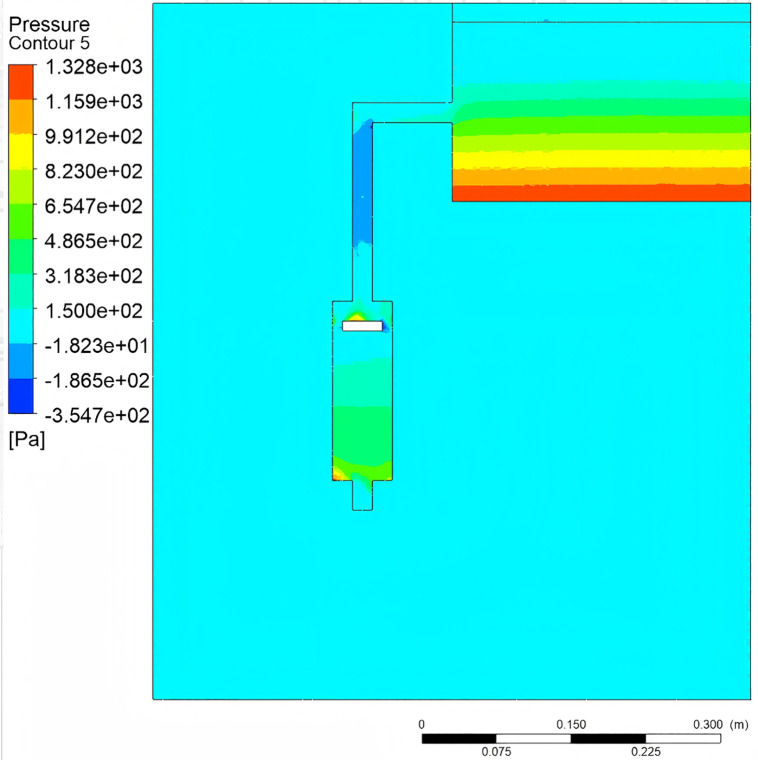
Pressure contour.

**Fig 20:**
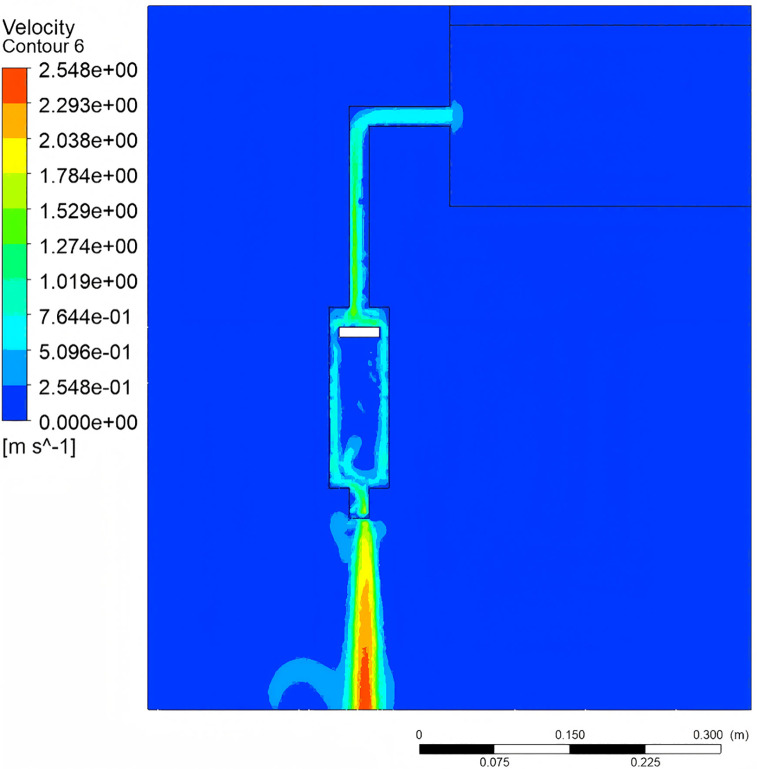
Velocity contour.

**Fig 21:**
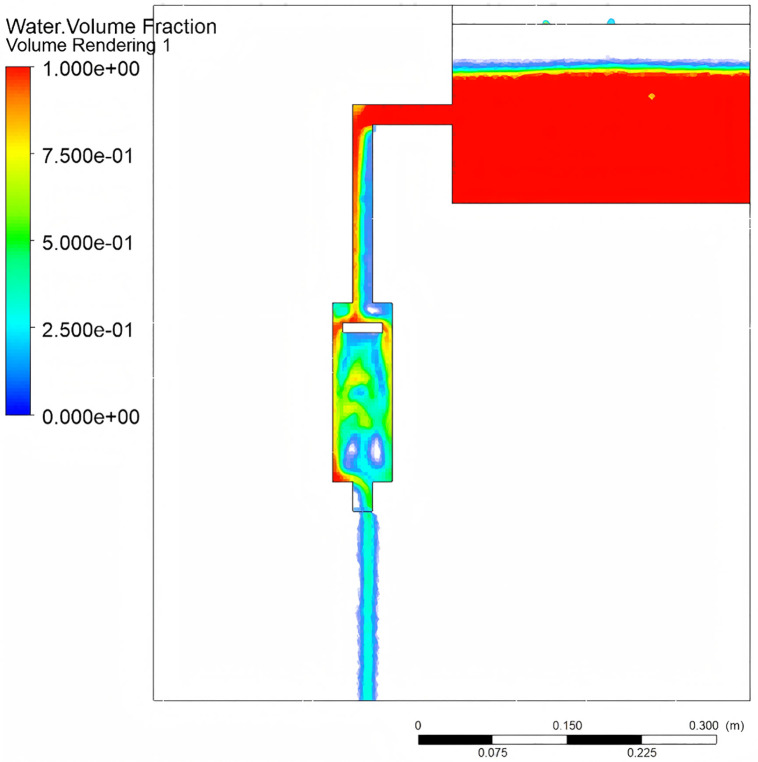
Liquid phase volume fraction plot.

The fluid dynamic behavior within the compression pump chamber is illustrated in Figs 19–21. When the crank angle reaches 180°, the piston attains top dead center, inducing a pressure rise in the upper chamber. Concurrently, an axial pressure gradient forms in the lower chamber due to the coupling of fluid compression and gravity, with the maximum pressure observed at the lowest extremity Fig 19. At the nozzle, the contraction of the flow passage cross-section yields a peak velocity of 2.54 m/s. In contrast, the flow velocity near the piston underside approaches zero, confirming that the piston motion effectively suppresses reverse flow Fig 21. Furthermore, as the water tank level decreases, the gas-liquid two-phase region in the upper pipeline exhibits a volume fraction of 0.92, with gas aggregation primarily governed by buoyancy Fig 21.

**Fig 22:**
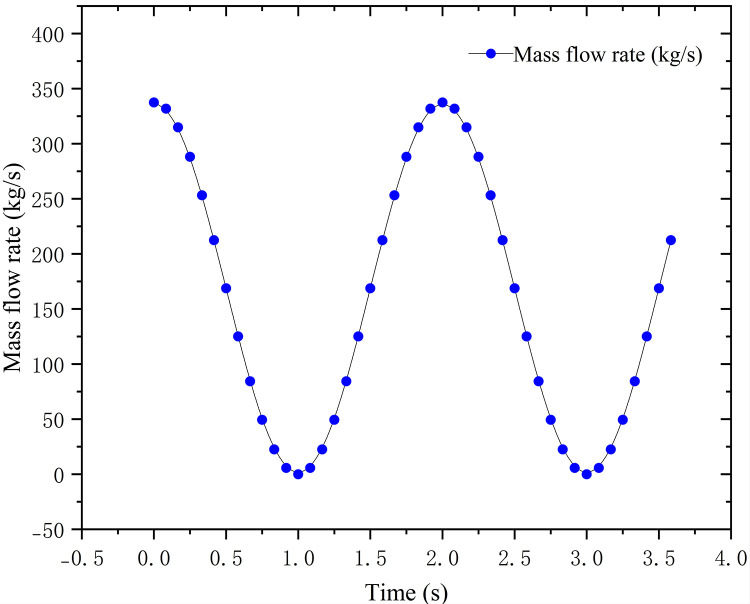
Mass flow rate of piston pump.

Fig 22 shows the mass flow rate variation diagram of the piston pump. The reciprocating motion of the piston causes periodic flow fluctuations in the chamber. During the discharge phase, the reciprocating motion induces a reverse inflow of −0.33755 kg/s at the lower end of the nozzle. The flow transitions to a forward direction during the upstroke, while the downstroke generates a negative flow with a single discharge of approximately 142.8 g of fluid, which aligns with the design expectations.

### 4.2 Seed dynamics simulation and seed metering device optimization

**Fig 23:**
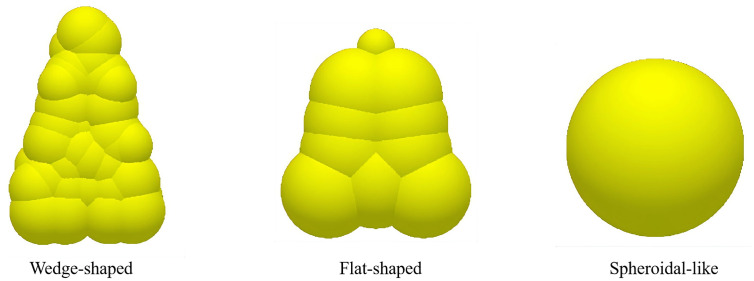
Corn seed model.

Based on the morphological characteristics of Zheng dan 958 maize seeds, a mixed particle model was constructed using the multi-sphere aggregation method, comprising 50% wedge-shaped, 30% flat-shaped, and 20% spherical-like particles, with a total of 900 particles, as illustrated in Fig 23. A discrete element model of the seed metering device was established on the EDEM simulation platform, employing the Hertz-Mindlin contact mechanics model [36]. The specific material parameters are listed in Tables 1 and 2

**Table 1:** Physical properties of corn seeds and seed metering devices.

Items	Poisson’s ration	Shear modulus/Pa	Density/(g.cm^-3^)
Corn seed	0.4	1.38 × 10^8^	1.197
Seed metering plate	0.34	3.0 × 10^9^	1.250
Alloy	0.33	2.63 × 10^8^	2.850

**Table 2:** Collision parameters between corn seeds and between corn seeds and seed metering devices.

Items	Static friction coefficient	Kinetic friction coefficient	Coefficient of restitution
With corn seeds	0.342	0.05	0.5
With seed metering plate	0.452	0.01	0.5
With the housing	0.386	0.08	0.65

Supplemental Simulation Parameter Settings: In EDEM, the time step was set to 20% of the Rayleigh time step, and the particle factory generation speed was 200 particles/s. In the CFD-DEM coupled simulation, the data exchange interval was 10 EDEM time steps, and the convergence criterion for the coupled calculation was a relative residual less than 10 ⁻ ³.

**Fig 24:**
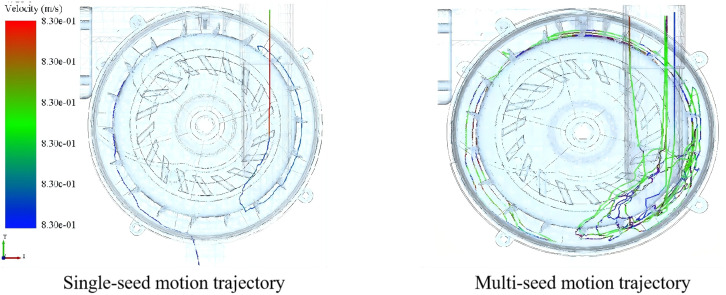
Seed displacement.

To gain an in-depth understanding of the dynamic behavior of seeds at different stages within the seed metering device, a CFD-DEM coupled simulation technique was employed to observe seed motion trajectories, as shown in Fig 24. The absence of seed pile-up in the figure is attributed to the simulation setup, which incorporated a seed filling rate 30% consistent with actual working conditions and an optimized seed agitation strip structure that ensures rapid dispersion and filling of seeds under gravity-dominated conditions. The seeds initially fill the seed plate rapidly under gravity-dominated conditions, followed by an orderly transition in motion state regulated by velocity gradients. Ultimately, they achieve precision seeding via an optimized low-speed parabolic trajectory. This process fully captures the dynamic evolution from high-speed seed filling to low-speed seed deposition [37].

**Fig 25:**
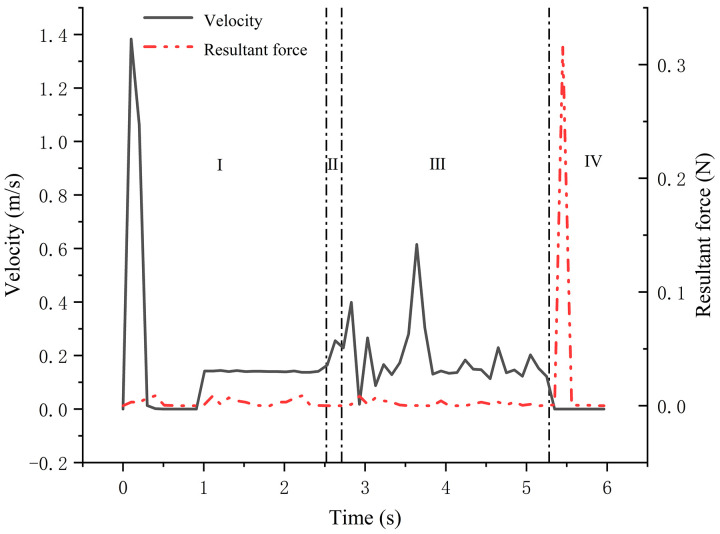
Variation of seed velocity and resultant force with time.

As shown in Fig 25, the resultant force and velocity variation of seeds were analyzed. During the gravity-filling phase (0-2.52 s), seed entered the cell wheel at 0.61 m/s, then decelerated abruptly to 0.0004 m/s through collisions for precise positioning. In the seed-clearing phase (2.52-2.71 s), seeds detached from cells at 0.15 m/s under combined shear and gravitational forces. Finally, during the centrifugal delivery phase (2.71-5.28 s), seeds accelerated to 0.61 m/s and were ejected with a parabolic trajectory at 0.130 ± 0.005 m/s. Through mechanism coordination, the coefficient of variation for ejection angle was reduced by 73.6% compared to conventional gravity-fed seed meters.

**Fig 26:**
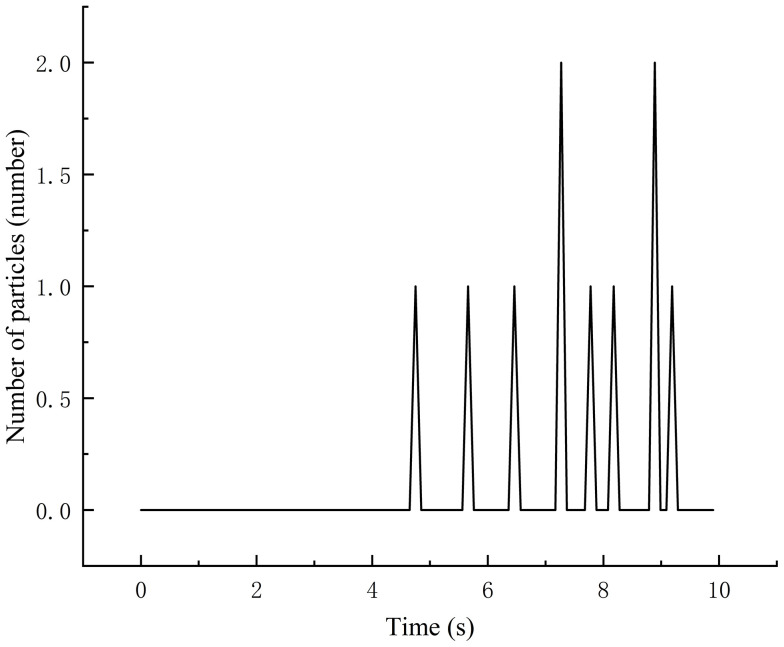
Number of particles in the area.

The simulation results show that the phenomenon of missed seeding and reseeding mainly occurs at 7.23 seconds and 8.91 seconds, as shown in Fig 26, and seeding is normal at other times. The qualified rate of seed sowing performance was 90.2%, and the rate of replanting was 3.73%. The curve is straight from 0 to 5.56 seconds, because no seed enters the seed discharge port during this time periods then, the seed outflow shows fluctuation distribution, reflecting the instability in the seeding process.

### 4.3 CFD-DEM coupling simulation of the broadcaster

To investigate the influence of the seed-guiding device on seeding performance, a CFD-DEM coupled simulation was employed to simulate the seed delivery process. Three structural configurations (A, B, and C) with variations in length (*l*), diameters ($d_{1}$, $d_{2}$), and flow channel geometry were designed to study the effects of different parameters on the seed-guiding mechanism, as detailed in Table 3. The contact parameters of the simulation materials are listed in Table 4.

**Table 3:** Parameters of different structure groups.

Group designation	*l*	*d*_*1*_	*d*_*2*_	θ
Group A	234.6	20	15	15
Group B	238.5	20	15	30
Group C	241.7	20	15	45

**Table 4:** Material properties and boundary conditions.

Parameters	Materials	
	Corn seeds	Seed metering device
Poisson’s ratio	0.4	0.33
Shear modulus	1.37E-08	2.60E + 10
Density (g/cm^3^)	1.321	2.85
Coefficient of restitution (with seeds)	0. 182	0.615
Static friction coefficient (with seeds)	0.432	0.386
Dynamic friction coefficient (with seeds)	0.0782	0.08

Connection to Overall Research Objectives: This simulation aims to identify the key structural parameters affecting zero-speed seed delivery performance, providing an optimization basis for the bench tests in Chapter 5 and ultimately realizing the zero-speed seeding theory proposed in Chapter 3.

#### 4.3.1 Analysis of simulation results.

**Fig 27:**
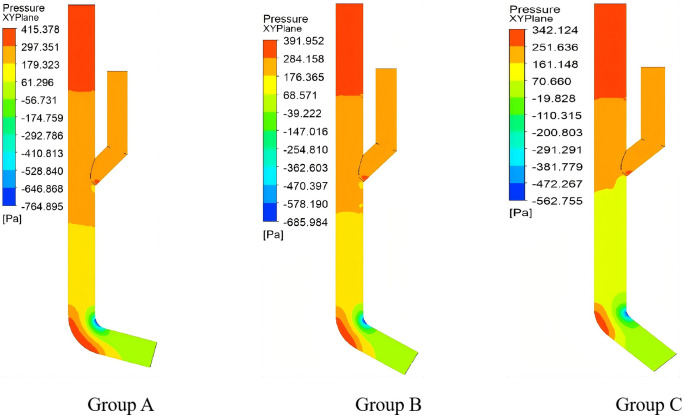
Pressure distribution in seed introduction device.

Flow Field Characteristics of the Seed Guide Device As shown in Fig 27, experimental group C demonstrated optimal performance in the seed guide device: A uniform pressure gradient formed between the mixing chamber and the guiding chamber, providing initial acceleration force for seeds while minimizing energy loss through moderate pressure differentials. In contrast, groups A and B exhibited excessive pressure differences, inducing turbulent seed motion. These results confirm the critical influence of pressure distribution on zero-velocity seed delivery performance.

**Fig 28:**
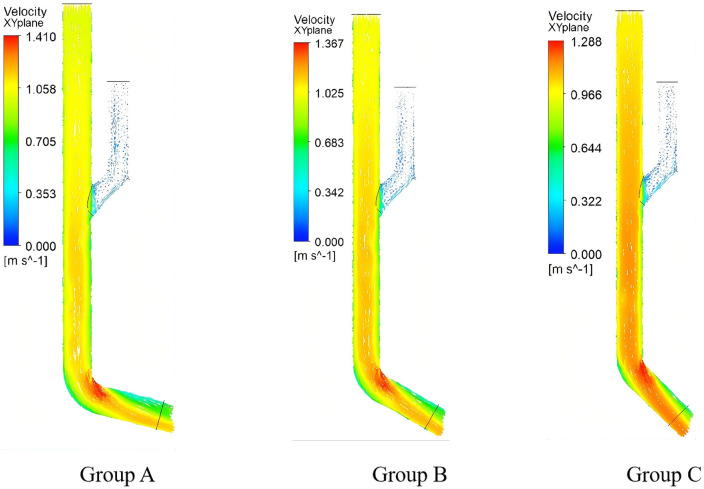
Distribution of water flow velocity in seed guiding device.

Velocity Profile Analysis of the Seed Guide Device As shown in Fig 28 illustrates that experimental group C achieved superior guiding performance: Its smooth velocity gradient ensured efficient seed transport while enabling terminal zero-velocity delivery. Groups A and B, however, displayed overly steep velocity gradients, leading to seed rebound and misalignment issues.

#### 4.3.2 Kinematic analysis of seed motion.

**Fig 29:**
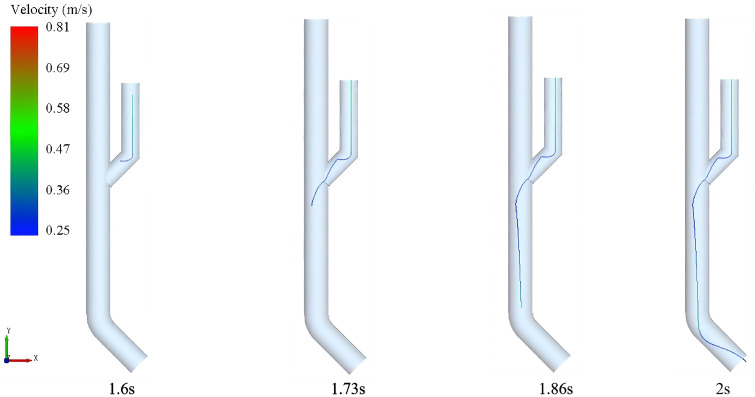
Seed movement track at different time in group C.

The study demonstrates that compared to Groups A and B, which exhibited disordered seed movement, the optimized Group C achieved balanced pressure gradients between the mixing chamber and seed delivery chamber. As shown in Fig 29, this design ensures effective seed acceleration while reducing delivery resistance, resulting in linear seed trajectories with minimal collisions. Consequently, the system attains highly efficient and precise control over the seeding process.

Simulation results indicate that when the seed pickup plate operates at 30 rpm with a cell depth of 8.2 mm and a filling angle of 60°, the seed metering device achieves optimal performance: a qualified seeding index of 90.2% and a multiple-seeding index of 3.73%. Concurrent fluid mechanism simulations demonstrate that the piston pump delivers a single-stroke discharge of 142.8 g, and when coupled with a 45°seed tube inclination angle, enables seed ejection velocity of 0.9 m/s – meeting precision planting requirements. These optimized parameters provide a theoretical basis for the experimental validation in Chapter 5, directly supporting the core objective of this research to achieve high-precision fluid hill-drop planting.

## 5. Bench experiment

### 5.1 Materials and methods

**Fig 30:**
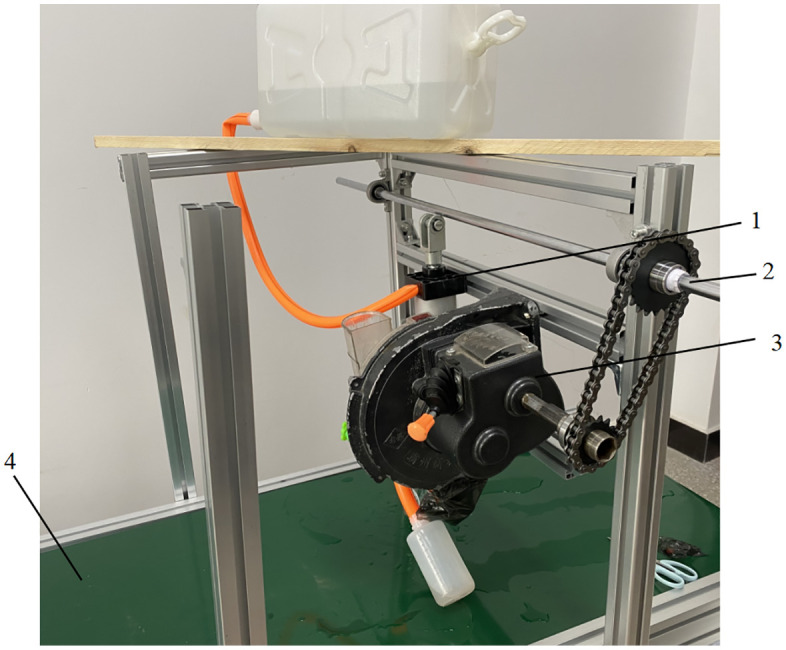
Test bench. 1. Fluid control mechanism; 2. Drive shaft; 3. Seed metering device; 4. Control console.

To validate the performance of the fluid-assisted hill-drop corn planter, a test platform was established in accordance with the GB/T 6973−2005 standard, as shown in Fig 30. The system comprised a fluid control mechanism, seed metering device, synchronous servo conveyor belt, and high-speed imaging system. Using Zheng dan 958 maize seeds and 3D-printed seed delivery tubes, key evaluation metrics included hill length, hill spacing, and fluid application error. Comparative experiments were conducted to assess the seeding performance of traditional spoon-wheel and disturbance-free strip-type seed metering devices.

### 5.2 Analysis of experimental results

#### 5.2.1 Hill-dropping performance evaluation.

**Fig 31:**
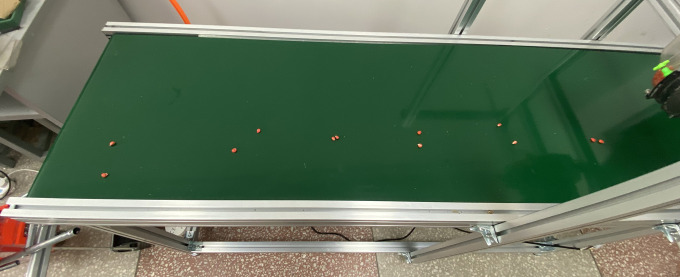
Seed metering effect of seed disturbing side-filling seed metering device.

**Fig 32:**
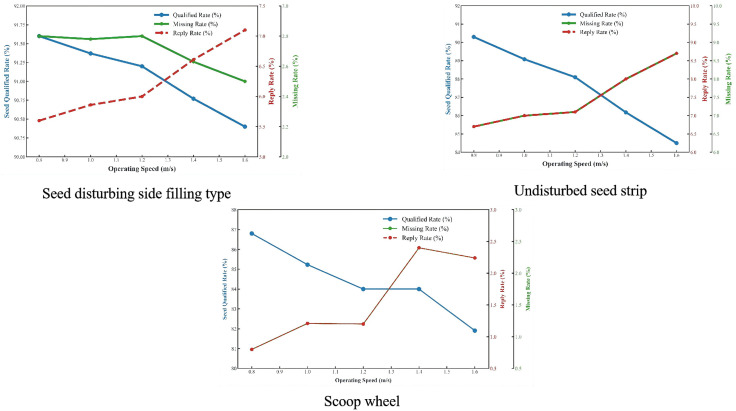
Comparison of seeding effect.

Based on theoretical analysis and simulation results, the optimized side-filling seed metering device with a scoop wall inclination angle of 60°and scoop radius of 7 mm demonstrated superior seeding performance, as shown in Fig 31. The seeding performance was evaluated according to GB/T 6973−2005, and a direct comparison was made against conventional seeders (spoon-wheel and non-agitating types) at speeds of 20, 30, and 40 rpm under identical bench conditions. Within the operational speed range of 0.8 ~ 1.6 km/h, the device achieved: Seedling Qualification rate>90%, Miss-seeding rate<2.8%, Multiple-seeding rate<9%. These metrics significantly outperformed conventional designs lacking seed-cleaning brushes and agitating strips (qualification rate <87%, miss-seeding rate >0.7%, multiple-seeding rate >12%), as illustrated in Fig 32. The experimental results validate that the agitating strip design and geometric optimization of seed scoops critically enhance filling efficiency while reducing the multiple-seeding index.

**Fig 33:**
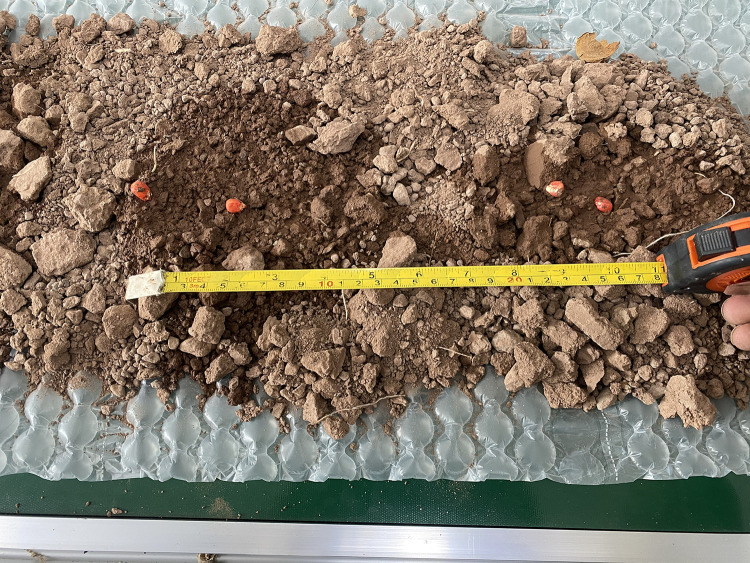
Measurement of hole-forming length.

**Fig 34:**
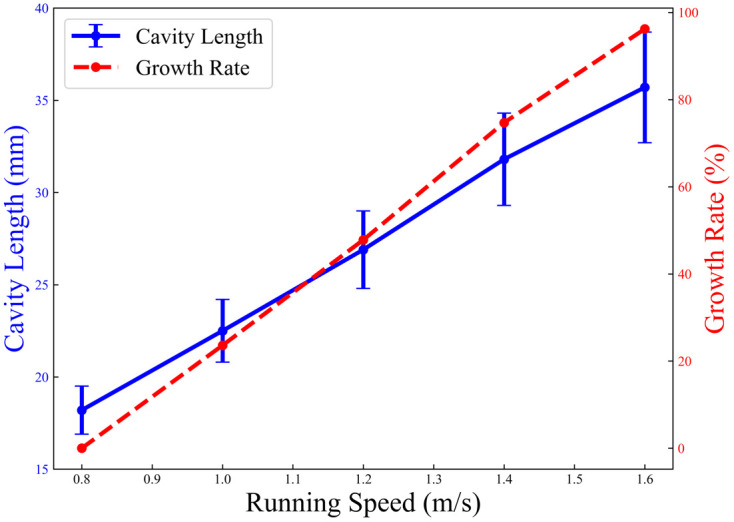
Characteristics of velocity and cavity length.

To validate the effectiveness of the zero-speed seeding technique in improving seeding accuracy, the effects of operating speed and soil hardness on hill length $L_{h}$ were investigated. Bench tests were conducted under controlled soil hardness conditions (0.5, 0.8, 1.2, 1.4, and 2.0 MPa) to simulate varied field environments. As shown in Figs 33 and 34, the measured hill length exhibited a quadratic relationship with operating speed ($L_{h}\infty v^{2}$) within the range of 0.8–1.6 m/s. Notably, under hard soil conditions (2.0 MPa), the hill length decreased by 42% compared to soft soil, indicating that soil plastic deformation is the dominant factor in energy dissipation during hill formation.

These findings are highly relevant to the core focus of this paper, as they quantitatively demonstrate how the zero-speed seeding mechanism—coupled with soil properties—affects the final seeding morphology. This provides critical insight into the operational adaptability of the proposed planter under varying field conditions, directly supporting the study’s objective of achieving precision planting in complex terrains.

**Fig 35:**
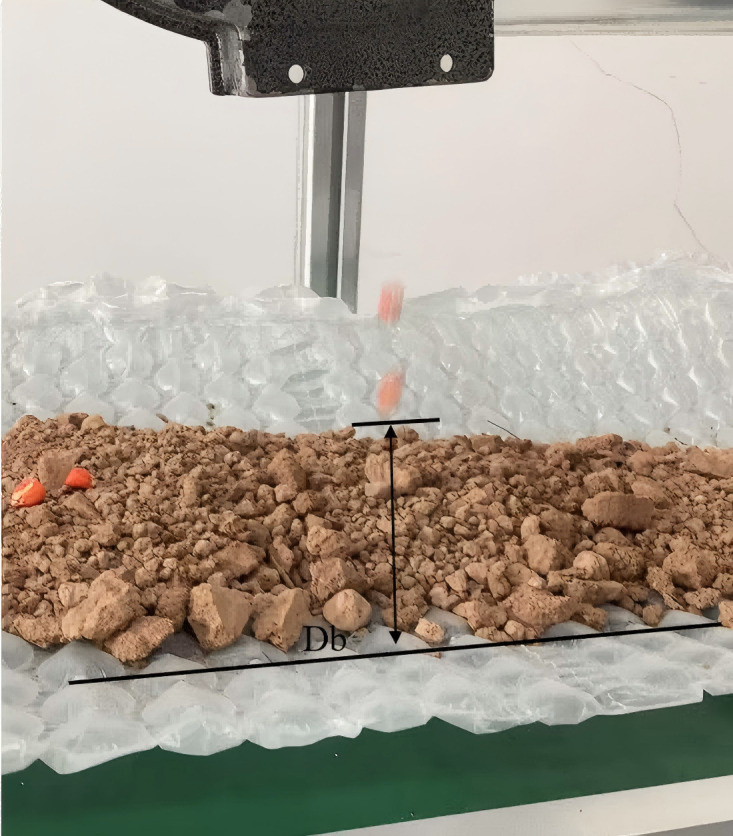
Seed bounce distance.

Fig 35 shows the seed bounce distance test. It can be seen from Table 5 that under the condition of 30 rpm speed of seed metering device, fluid seeding technology can effectively inhibit seed bouncing, realizing zero-speed seed throwing ratio ($P_{0}$) of 92.3%(soft soil) and 85.7%(hard soil), and the average bouncing distance of non-zero-speed seeds ($D_{b}$ =12.5 mm) is significantly greater than that under zero-speed condition (2.1 mm), which fully proves the key role of speed-speed coordinated control on seeding accuracy.

**Table 5:** Seed bounce distance.

Soil hardness S(MPa)	Zero-speed ratio P_0_(%)	Average bounce distance Db(mm)
0.5	92.3	2.1
0.8	90.5	2.9
1.2	88.6	3.8
1.6	86.8	4.9
2.0	85.7	5.4

The results show that zero-speed seeding technology can reduce the bounce distance to less than 5.4 mm and improve the uniformity of seedlings in hard soil.

#### 5.2.2 A study on discharge of each hole.

**Fig 36:**
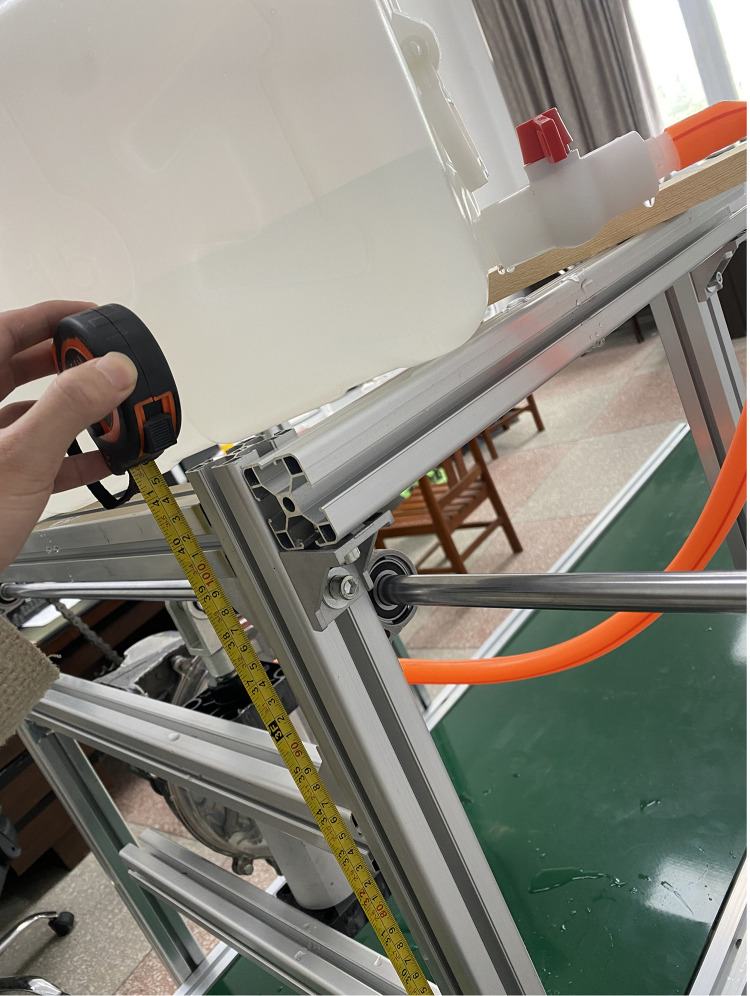
Tank height measurement.

To study the influence of water level height of water tank and operating speed of unit on water quantity of hole. The rotation speed of the seed metering device is adjusted to 11.2, 15.6 and 20.1 rpm through the motor reduction gear to simulate the low-speed operation of the tractor. The height of the water tank is 1.5 m and 1.2 m. The water application data is collected by hanging water bottles as shown in Fig 36 to reveal the correlation law between the operation parameters and the water application performance.

**Fig 37:**
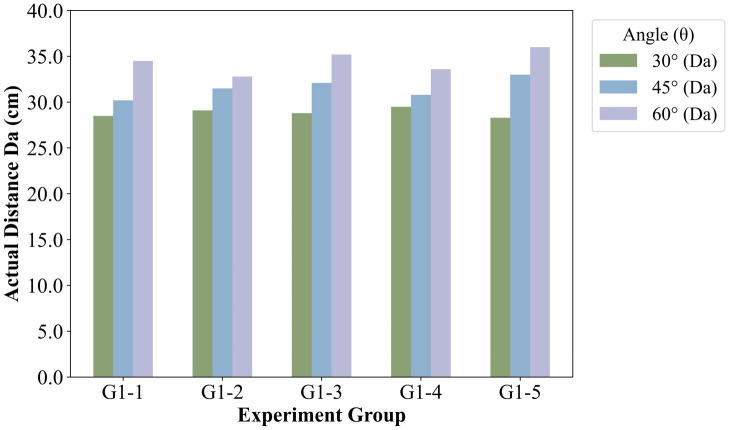
Effects of different seeding parameters on hill-drop water application volume.

As shown in Fig 37, When the speed of the seed metering device increased from 11.2 r/min to 20.1 r/min, the water application per hill increased significantly by 13.7%. In contrast, variation in water tank height within the range of 1.2 ~ 1.5 m resulted in only a 2% fluctuation in water application. This result verifies that the dynamic compensation effect of the plunger piston mechanism effectively mitigates the influence of hydrostatic head variation, thereby significantly improving watering stability. It demonstrates that the speed of the seed metering device (and thus the pumping frequency) is the dominant factor regulating water application, while changes in water tank height within a reasonable range have no significant impact on outflow uniformity.

#### 5.2.3 Influence of seed guide tube inclination on hill spacing accuracy.

**Fig 38:**
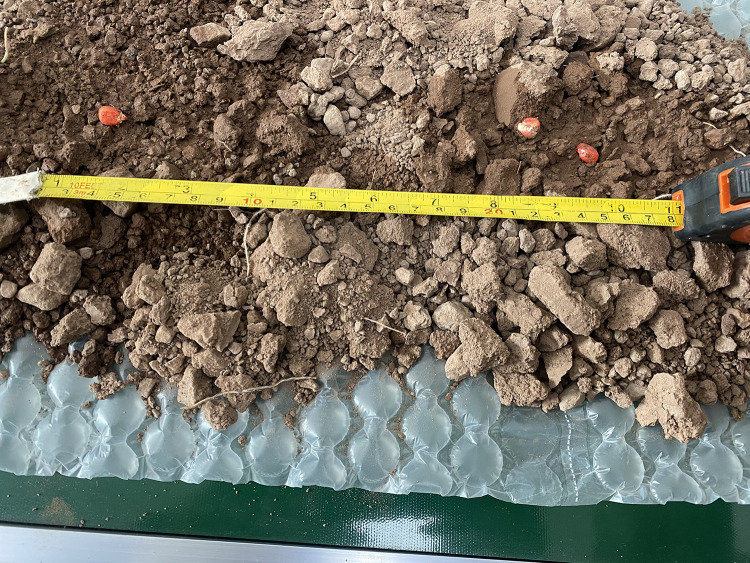
Hole spacing measurement.

The inclination angle of the seed guide tube is a critical parameter governing the seed trajectory and directly influences hill spacing uniformity. To evaluate its effect, bench tests were conducted using a 3D-printed ABS resin seed guide tube. The tests were performed at a constant seed-metering device speed of 20 rpm, with the tube inclination angle (θ) set to 30°, 45°, and 60°. Hill spacing deviation (ΔD) was measured as shown in Fig 38.

**Fig 39:**
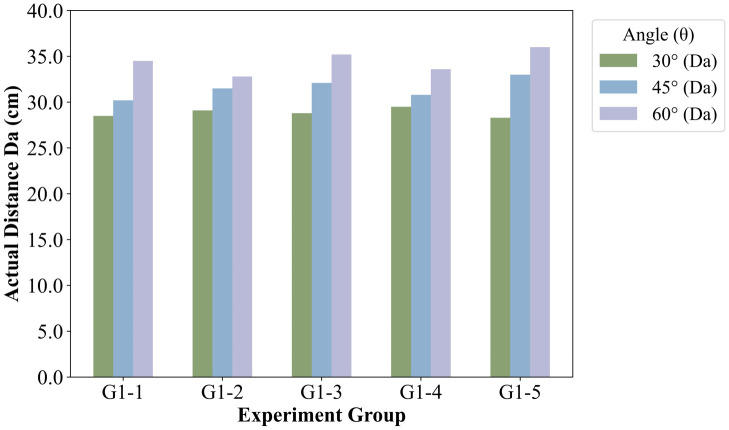
Effect of seed guide tube inclination angle on hill-drop spacing.

As illustrated in Fig 39, five replicate tests for each inclination angle demonstrated that the inclination angle significantly affects seeding spacing accuracy. An optimal inclination range of 30°–40° was identified, within which ΔD could be controlled within 5%. This optimal range ensures stable seed discharge with minimal lateral velocity, corroborating the simulation predictions regarding the zero-speed delivery performance. The results confirm that the seed guide tube inclination is a key factor determining hill spacing accuracy, directly impacting the realization of the zero-speed seeding theory.

#### 5.2.4 Evaluation of seed-water coincidence rate.

**Fig 40:**
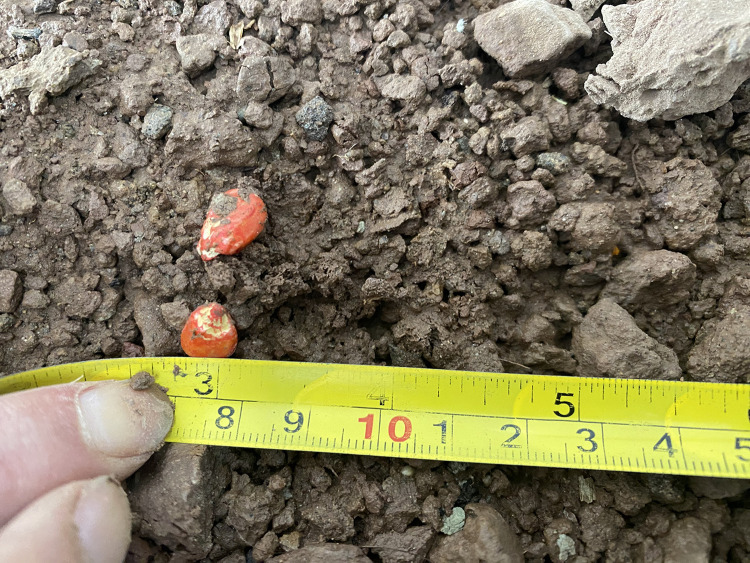
Measurement of the coincidence rate of 4 kinds of acupoints.

The synchronization of seed and water placement is crucial for effective fluid seeding. As shown in Fig 40, To evaluate this, the seed-water coincidence rate (Ra) was investigated under different operational parameters. The seed guide tube inclination was fixed at 45°, and tests were conducted over a continuous sowing distance exceeding 10 meters. A seed was considered coincidence if it landed within ±3.5 cm of the center of a hydrated hill. The conveyor belt speed was varied between 0.8 m/s and 1.6 m/s, with a theoretical hill spacing of 30 cm. The performance under different seed-metering device speeds (20, 30, 40 rpm) and conveyor belt speeds is summarized in Table 6. The results demonstrated that the seed-water coincidence rate exceeded 89% under multiple parameter combinations, fulfilling agronomic requirements. A particularly high coincidence rate of 94.8% was achieved with a seed-metering device speed of 30 rpm and a conveyor belt speed of 1.2 m/s, which also corresponded to a superior hill spacing accuracy of ΔD ≤ 1.0%. This specific parameter combination (T5 in Table 6) proves to be optimal for synchronizing seed and fluid delivery, ensuring both high coincidence and precise hill spacing. These findings provide critical guidance for optimizing the operational parameters of high-performance fluid-based hill-drop drilling systems.

**Table 6:** Different experimental groups.

Experimental group	Seed-metering device speed (rpm)	Conveyor belt speed (m/s)	Actual hill spacing Da (cm)	Hill spacing deviation ΔD (%)	Seed-water coincidence rate Ra (%)
T1	20	0.8	29.2 ± 0.8	2.7	88.5 ± 2.1
T2	20	1.2	34.5 ± 1.5	15	62.3 ± 3.8
T3	20	1.6	38.1 ± 2.1	27	48.6 ± 4.5
T4	30	0.8	28.9 ± 0.6	3.7	91.2 ± 1.7
T5	30	1.2	30.3 ± 0.5	1	94.8 ± 1.2
T6	30	1.6	32.7 ± 0.9	9	79.4 ± 2.4
T7	40	0.8	27.5 ± 1.2	8.3	73.6 ± 3.0
T8	40	1.2	29.8 ± 0.7	0.7	89.1 ± 2.3
T9	40	1.6	31.1 ± 1.0	3.7	85.2 ± 2.6

## 6. Conclusion

This study designed, optimized and validated a novel precision fluid hill-drop planter that integrates fluid-control mechanisms with zero-speed seeding theory. Empirical verification revealed bench test performance with a seeding qualification rate exceeding 95%, a reseeding rate below 9%, a seed-hole collocation rate of 94.8% in field-performance tests, and a reduction in seed-bouncing distance by 63.2% relative to conventional methods. The system-maintained hole-spacing deviations under 1.0% and fluid-application error per hole below 2%, thereby achieving high spatial accuracy and input precision. The core innovation lies in the synergistic application of fluid control and zero-speed seeding theory, guided by CFD–DEM simulation and validated experimentally (with a 4.8% simulation-to-experiment error). These results confirm both the technical robustness and agronomic potential of the approach. Ultimately, the research provides an effective and reliable technical solution for mechanizing maize planting in complex terrains, directly addressing the limitations of existing equipment related to terrain adaptability and seeding precision. By significantly enhancing seeding qualification, reducing seed displacement and ensuring precise fluid application, the technology offers a practical pathway to improve crop establishment and water-use efficiency, thereby supporting sustainable agricultural development in hilly regions of Southwest China and beyond.

## 7. Discussion

This study confirms that the developed precision fluid hill-drop planter achieves highly reliable seed placement under controlled bench testing. The consistent seed depth and spacing validate the efficacy of the mechanical design and operational regime, which are widely recognized as critical determinants of uniform germination and early stand establishment. Compared with conventional mechanical seeders, the system demonstrates marked performance improvements: a seeding qualification rate above 95% and a seed-bounce distance reduced to approximately 5.4 mm—substantially outperforming the qualification rates below 90% and bounce distances exceeding 15 mm typically reported for conventional systems under similar conditions.

Unlike most existing research focused primarily on flat-terrain optimization, this work explicitly addresses the technological gap in precision seeding for sloped or uneven topographies, thereby establishing a clear contextual innovation. The core achievement lies in the successful integration of fluid control with zero-speed seeding theory, a synergy guided by CFD–DEM coupling simulation and robustly validated by the low simulation-to-experiment relative error of 4.8%. Although the current validation was conducted on horizontal test benches, the results provide a solid performance baseline and verify the fundamental mechanics essential for future terrain-adaptive development.

The significance of these findings is substantial. This research provides a practical pathway to extend precision planting technology into hilly agricultural landscapes, contributing directly to mechanization in challenging terrain. The simultaneous enhancement of seeding precision and water-use efficiency, as evidenced by the high seed-hole collocation rate and minimal fluid application error, promises to improve crop establishment and support sustainable agricultural development in underserved regions like Southwest China. It is acknowledged that this study, while establishing technical feasibility, did not encompass long-term agronomic evaluation or performance quantification across slopes, which are necessary steps toward practical deployment.

Consequently, future work will follow two complementary paths: large-scale comparative field trials to assess full agronomic impact and operational efficiency, alongside dedicated research into slope-adaptation. The latter will involve quantitative performance characterization across gradients and the development of an adaptive control system with real-time feedback, ultimately guiding the technology from prototype validation toward field-scale adoption.
